# Evaluating and Validating the Fluorescent Probe Methodology for Measuring the Effective Hydrophobicity of Protein, Protein Hydrolyzate, and Amino Acid

**DOI:** 10.1021/acs.jafc.4c07664

**Published:** 2024-11-30

**Authors:** Nattawan Chorhirankul, Anja E.M. Janssen, Remko M. Boom, Julia K. Keppler

**Affiliations:** Food Process Engineering Group, Wageningen University, P.O. Box 17, Wageningen 6700 AA, The Netherlands

**Keywords:** hydrophobicity, fluorescent probe, ANSA, PRODAN, protein, protein hydrolyzate, amino acid

## Abstract

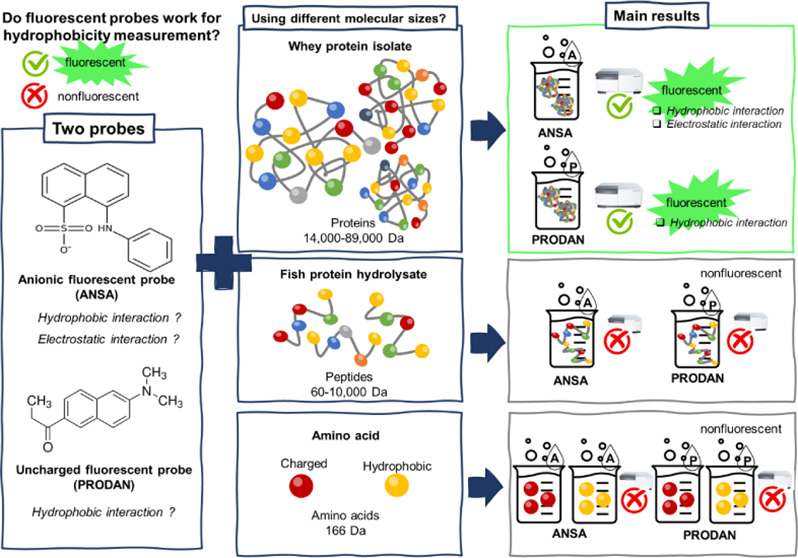

The fluorescent probe method with 8-anilino-1-naphthalenesulfonic acid ammonium salt (ANSA) and 6-propionyl-2-(*N*,*N*-dimethylamino) naphthalene (PRODAN) was validated to determine the effective hydrophobicity of the whey protein isolate. The focus was on charge and hydrophobic interactions due to the complexation between the proteins and probes. Using ANSA could overestimate the effective hydrophobicity of positively charged proteins. Furthermore, the relative fluorescence intensities (RFIs) should be considered before determining the effective hydrophobicity by linear regression. This is to be confident that the obtained RFI mainly originates from the hydrophobic interaction. The validated protocol was then applied to protein hydrolyzate and amino acids to investigate the method’s reliability for small molecules. Adding ANSA or PRODAN probes to solutions containing protein hydrolyzates (60–10,000 Da), or the amino acids, tryptophan, glutamic acid, and lysine (∼165.85 Da), did not affect RFI. The effective hydrophobicity of those small constituents, therefore, could not be determined by these probes.

## Introduction

Hydrophobicity, or the dislike of water, is one of the molecular properties that significantly influence the functionality of food ingredients. Amphiphilic macromolecules such as proteins typically have more hydrophobic regions than other regions. Their surface or effective hydrophobicity has been intensively studied as it relates to their three-dimensional structure, which determines their stability and functional properties such as foaming, emulsifying, and gelling.^1−3^ As many hydrophobic regions in intact proteins are not on their surface but inside, partial hydrolysis of these proteins into peptides that consist of 2–20 amino acid residues can result in exposing hydrophobic side chains.^4^ Relatively hydrophobic peptides are, by definition, not highly soluble and, therefore, could negatively influence processing, e.g., membrane separation. Bouhallab and Henry^5^ reported that the high rejection of an inorganic ultrafiltration membrane for a hydrolyzate was caused by hydrophobic interaction between hydrophobic peptides and the used membrane support. Lapointe et al.^6^ also found that the hydrophobic interaction between β-lactoglobulin tryptic peptides and a nanofiltration membrane showed evidence of fouling, which affected the selective properties of the membrane during separation. The characterization of the hydrophobicity of proteins and peptides is therefore crucial not only for understanding and controlling their (bio)functionality but also for their processing. However, quantitative analysis of the hydrophobicity without knowing the molecular structure or using powerful techniques, such as hydrophobic interaction chromatography (HIC) and reversed-phase high-performance liquid chromatography (RP-HPLC), is challenging and still unreliable for protein or peptide mixtures.

Fluorescent probes are widely used to quantify the effective protein hydrophobicity. Fluorescence involves excitation by irradiation at a certain wavelength followed by the measurement of emitted radiation at a longer wavelength.^7^ Suitable probes typically have a low fluorescence yield in aqueous solution, which increases after binding with hydrophobic surface areas of proteins.^8^ As a result, the emission intensity typically relates to the protein’s surface hydrophobicity. This allows us to quantify the average hydrophobicity of protein or peptide mixtures without specific information about their structures. The fluorescent probe method is supposed to have high selectivity and sensitivity and is simple and relatively inexpensive.^7−9^ Nevertheless, the method depends on the binding between the probe and hydrophobic regions of molecules in a mixture, which is also a function of their structure. The fluorescent probe method is also applied for quantification of the hydrophobicity of protein hydrolyzates, but for this, it is considered a qualitative technique. Validation for these shorter-chain molecules is scarce.

8-Anilinonaphthalene-1-sulfonic acid (ANS) and 8-anilino-1-naphthalenesulfonic acid ammonium salt (ANSA) are the most popular anionic fluorescent probes^9^ for the measurement of protein hydrophobicity. Even though these anionic probes have a hydrophobic group that binds to the hydrophobic areas of proteins, the charge is likely to also contribute to the interaction.^8,9^ Therefore, the hydrophobicity measured by this anionic probe can be wrong, especially for proteins with many charged surface areas. To avoid this charge effect, an uncharged aromatic probe, 6-propionyl-2-(*N*,*N*-dimethylamino) naphthalene (PRODAN), was introduced. The influence of charge interaction due to changing pH on the measurement of protein hydrophobicity has been revealed in a few studies. Haskard and Li-Chan^9^ evaluated the surface hydrophobicity of bovine serum albumin and ovalbumin with ANS and PRODAN and found that the charge interaction cannot be neglected. The ionic strength had a significant impact on the surface hydrophobicity of bovine serum albumin measured with both ANS and PRODAN. The surface hydrophobicity of ovalbumin measured by ANS increased with the ionic strength but did not change when PRODAN was used instead of ANS. Alizadeh-Pasdar and Li-Chan^8^ compared the surface hydrophobicity of native and heated proteins at different pHs using anionic and uncharged fluorescent probes. They showed that the surface hydrophobicity of all proteins was lowest at pH 3 using PRODAN measurements but had the highest value at the same pH with ANS. This confirms the importance of charge interaction between the anionic probe and proteins.

Anionic and uncharged fluorescent probes clearly provide different perspectives on protein hydrophobicity as they have different charge interactions. However, this has not yet been investigated for shorter-chain peptides. We hypothesize that given the larger configurational mobility of the shorter-chain peptides and the subsequent larger entropic effect of the association, the binding interaction between probes and peptides or amino acids may be weaker than that with proteins, while the charge interaction may be stronger. Therefore, this study compares an anionic (ANSA; MW ∼ 316.37 Da) and a nonionic fluorescent probe (PRODAN; MW ∼ 227.30 Da) for the determination of the effective hydrophobicity of whey protein isolate (WPI; MW ∼ 14,000–89,000 Da) with the aim of validating the fluorescent probe method and confirming the role of charge interaction at various pH values. The validated protocol was then applied to solutions of a fish protein hydrolyzate (Prolastin; MW ∼ 60–10,000 Da) and three individual amino acids (Trp, Glu, Lys; average MW ∼ 165.85 Da) to evaluate the reliability of fluorescent probe methods for mixtures of smaller molecules and to characterize their effective hydrophobicity.

## Materials and Methods

### Materials

**Figure 1:**
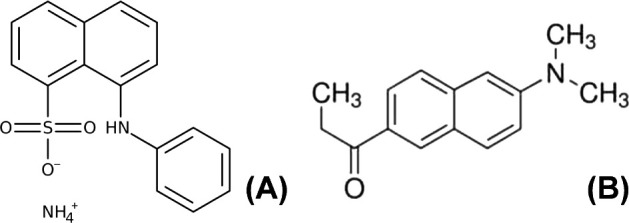
Chemical structure of fluorescent probes: (A) ANSA and (B) PRODAN.

Whey protein isolate (WPI-BiPro) lot no. JE 034–7–440–3 with a protein content of 97.9% (dry basis) and Prolastin (fish protein hydrolyzate) lot no. 220515A with a protein content of 88.5% (w/w) were provided by NIZO food research B.V. (Ede, Netherlands) and Copalis (Le Portel, France), respectively. L-Tryptophan (HPLC Reagent grade, ≥98%), l-glutamic acid (HPLC ReagentPlus*, ≥99%), and l-lysine (Food grade, ≥98%) were purchased from Sigma-Aldrich (Steinheim, Germany). Two fluorescent probes, 8-anilinonaphthalene-1-sulfonic acid ammonium salt (ANSA, 98%) and *N*,*N*-dimethyl-6-propionyl-2-naphthylamine (PRODAN, HPLC BioReagent, ≥98.0%), were obtained from Thermo Fisher Scientific (USA) and Sigma-Aldrich (Steinheim, Germany), respectively. The chemical structures of both probes are illustrated in Figure 1. The average molecular weight and isoelectric point values of the materials were summarized, as shown in Table 1.

**Table 1:** Molecular Weight and Isoelectric Point of Materials

Material	Molecular weight (Da)^11−14^	Isoelectric point^11−16^
Whey protein isolate	14,000–89,000	<5.5
β-Lactoglobulin (>50%)^14,17^	18,200–18,300	5.3–5.5
α-Lactalbumin (>20%)^14,17^	14,000	4.2–4.5
Bovine serum albumin (∼5–10%)^14,17^	65,000–69,000	4.1–5.5
Prolastin	60–10,000	5
L-Tryptophan	204.23	5.89
l-Glutamic acid	147.13	3.22
l-Lysine	146.19	9.74
ANSA	316.37	-
PRODAN	227.30	-
Citric acid	192.12	-
Na_2_HPO_4_	141.96	-

McIlvaine or citrate-phosphate buffer was prepared according to McIlvaine.^10^ The buffer solutions at pH 3–8 were mixtures of 0.1 M citric acid (Sigma-Aldrich, USA) and 0.2 M Na_2_HPO_4_ (Sigma-Aldrich, Germany) at different ratios. For the pH 9 buffer, the buffer at pH 8 was prepared and then adjusted by adding 1 M NaOH (Sigma-Aldrich, Germany). All buffers were freshly prepared before use.

### Preparation of Stock and Protein Solutions

The protocol mainly followed that of Alizadeh-Pasdar and Li-Chan.^8^ WPI, Prolastin, and the three amino acids were dissolved in Milli-Q water to make stock solutions with a concentration of 0.5% (w/v). To obtain the desired pHs (3, 5, 7, 8, and 9), the stock solutions were adjusted by adding 2 M NaOH or 2 M HCl (Sigma-Aldrich, Germany). The stock solutions were then serially diluted by specific buffers to obtain protein solutions with concentration ranges of 0.005–0.025% (w/v) or 0.05–0.25% (w/v) and 0.002–0.01% (w/v) for measurements using ANSA and PRODAN, respectively. The ANSA stock solution with a concentration of 8 mM was prepared by dissolving ANSA in 0.1 M phosphate buffer (pH 7.4). The PRODAN stock solution at 1.41 mM was obtained by mixing PRODAN in methanol (Actu-All Chemicals, Netherlands). The ANSA stock solution was stored in a DURAN bottle covered with aluminum foil to prevent light effects at room temperature. The PRODAN stock solution was kept in a DURAN bottle sealed and covered with Parafilm and aluminum foil to avoid evaporation and light reactions. The PRODAN stock solution was stored in a freezer (≤−10 °C) before use. The stock solution was held in an ice bucket when doing experiments.

### Measurement of the Relative Fluorescence Intensity (RFI)

The sample set was prepared by adding 20 μL of ANSA or 10 μL of PRODAN stock solution to 4 mL of protein (protein, hydrolyzate, or amino acid) solutions. Then, the sample mixtures were mixed by a vortex and kept in the dark for 15 min but no longer than 30 min at room temperature. The RFI of each solution was measured at room temperature with a Shimadzu RF6000 Fluorimeter (Kyoto, Japan) by using quartz cuvettes (Hellma Analytics, Müllheim, Germany). All cuvettes were rinsed using Milli-Q water, followed by ethanol 96% vol (VWR Chemicals, France) and dried with nitrogen gas before use. Excitation and emission wavelengths were set at 390 and 470 nm for the ANSA measurement and 365 and 465 nm for the PRODAN measurement, respectively. The slit widths of all wavelengths were 5 nm. The RFI for each solution was measured in triplicate. The controls consisted of protein solutions without adding any fluorescent probe stock solutions. The RFI of each control solution was measured in the same way as indicated before. To control the fluorimeter fluctuation due to different experimental days, the RFI of 4 mL of methanol with 10 μL of ANSA or PRODAN stock solution was measured for standardization. All experiments were performed in duplicate.

### Calculation of the Net RFI and Determination of the Effective Hydrophobicity

The net RFI of a protein solution at a specific concentration was calculated by subtracting the RFI of each control solution (the protein solution without adding fluorescent probes) from the value of the corresponding protein solution with ANSA/PRODAN. The linear slope of plotting between net RFIs and concentrations was indicated as the effective hydrophobicity of such a protein/protein hydrolysate/amino acid.

### Fluorescence Emission Scans of PRODAN

Proteins (WPI, Prolastin, tryptophan, lysine, and glutamic acid) were dissolved in McIlvaine buffer (pH 7) at 0.025%, 0.25%, and 0.5% (w/v) to make protein solutions. The PRODAN stock solution was added to 4 mL of the protein solution and mixed by a vortex. The mixture was left to stand in the dark for 15 min at room temperature. After that, the sample was measured at a fluorescence emission wavelength between 400 and 600 nm using a Shimadzu RF6000 Fluorimeter (Kyoto, Japan) with an excitation wavelength of 365 nm and a scan speed of 60 nm/min. All control solutions (without PRODAN) were scanned under the same conditions as described. All experiments were duplicated.

## Results and Discussion

### Using Fluorescent Probes with WPI

**Figure 2:**
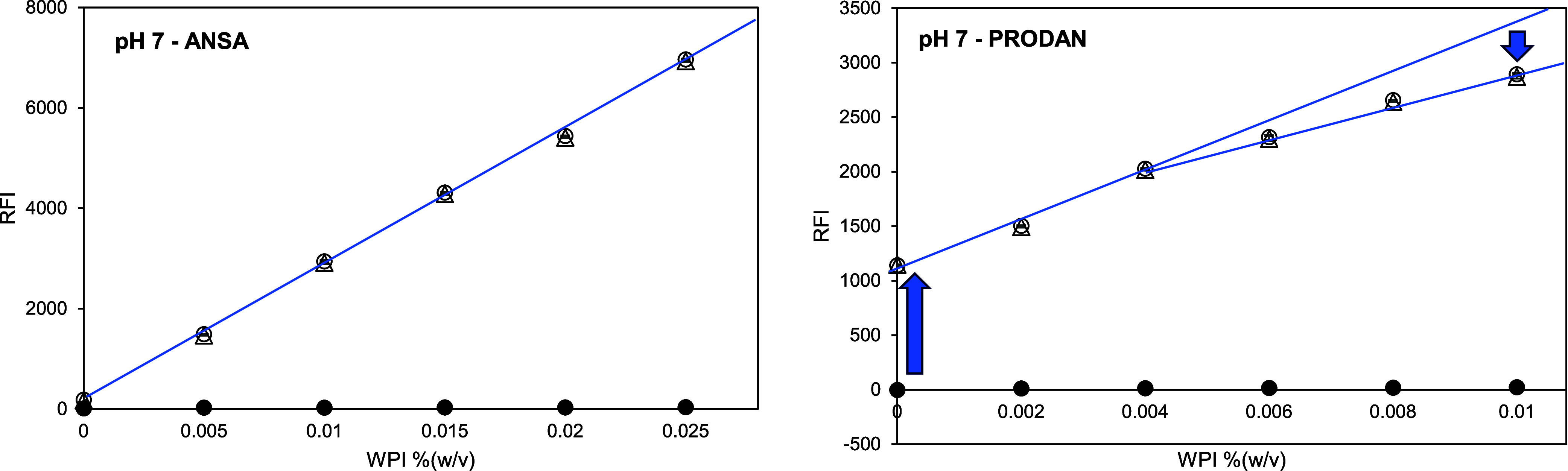
Relative fluorescence intensity (RFI) of whey protein isolate (WPI) in McIlvaine buffer at pH 7 and 8 mM ANSA or 1.41 mM PRODAN in WPI solution at pH 7 versus WPI concentration (between 0.002 and 0.025% (w/v) or approximately 0.002–0.02 mM). Open symbols represent the RFI of a WPI solution with ANSA or PRODAN. Closed symbols represent the RFI of a WPI control solution without ANSA or PRODAN. Triangles represent the net RFI of a WPI solution, which is the result of subtracting the RFI of WPI control solution from the RFI of WPI solution with ANSA/PRODAN. Excitation/emission wavelengths for ANSA and PRODAN were 390/470 nm and 365/465 nm, respectively. The blue dotted line is for a guide for the eye, and blue arrows indicate attention points discussed in the text. Note: the difference in WPI concentrations for ANSA and PRODAN measurements is due to different fluorescent properties.

The relative fluorescence intensities (RFI) of the WPI control solutions (without fluorescent probes) and WPI solutions with ANSA and PRODAN at various concentrations and pH 7 are shown in Figure 2. The results at the other pH values are shown in Figures S1–S2. Additionally, the net RFI values for WPI solutions are shown, which are the difference between the RFI values of the WPI solution with ANSA/PRODAN and with the WPI control solutions. The net values did not deviate from the RFI estimates of the WPI solutions with ANSA/PRODAN (Figure 2). This is because the RFIs of WPI control solutions without probes were considerably lower than those of WPI solutions with the probes.

The data points at 0% (w/v) WPI were the RFI or net RFI information on McIlvaine buffer (Figure 2). For ANSA, the RFI of the buffer with ANSA and its net RFI were close to 0, indicating no interaction between ANSA (MW ∼ 316.37 Da) and citric acid (MW ∼ 192.12 Da) or Na_2_HPO_4_ (MW ∼ 141.96 Da) in the buffer. The relatively low self-association of ANSA is supported by the dependence of the fluorescence intensity of ANSA on the polarity of the solvents. Slavík^18^ and Möller and Denicola^19^ reported that the intensity of ANS decreased with increasing the polarity of solvents (e.g., *n*-octanol, *n*-butanol, *n*-propanol, ethanol, methanol, ethylene glycol, and water) over the emission wavelength between 380 and 580 nm. They also show that the fluorescence emission was also approximately 0 when ANS was in water. For the uncharged PRODAN, the RFI without protein was already quite high with 1100 RFI. This is in line with previous findings, reporting that the presence of a polar aqueous environment causes a reduction in the conformational motion of PRODAN, predominantly stabilizing a planar, fluorescence emitting conformation.^20−22^ As the PRODAN emission spectrum is quite complex, it is recommended not only to study the RFI at a fixed wavelength but also to consider the whole emission spectrum.

**Figure 3:**
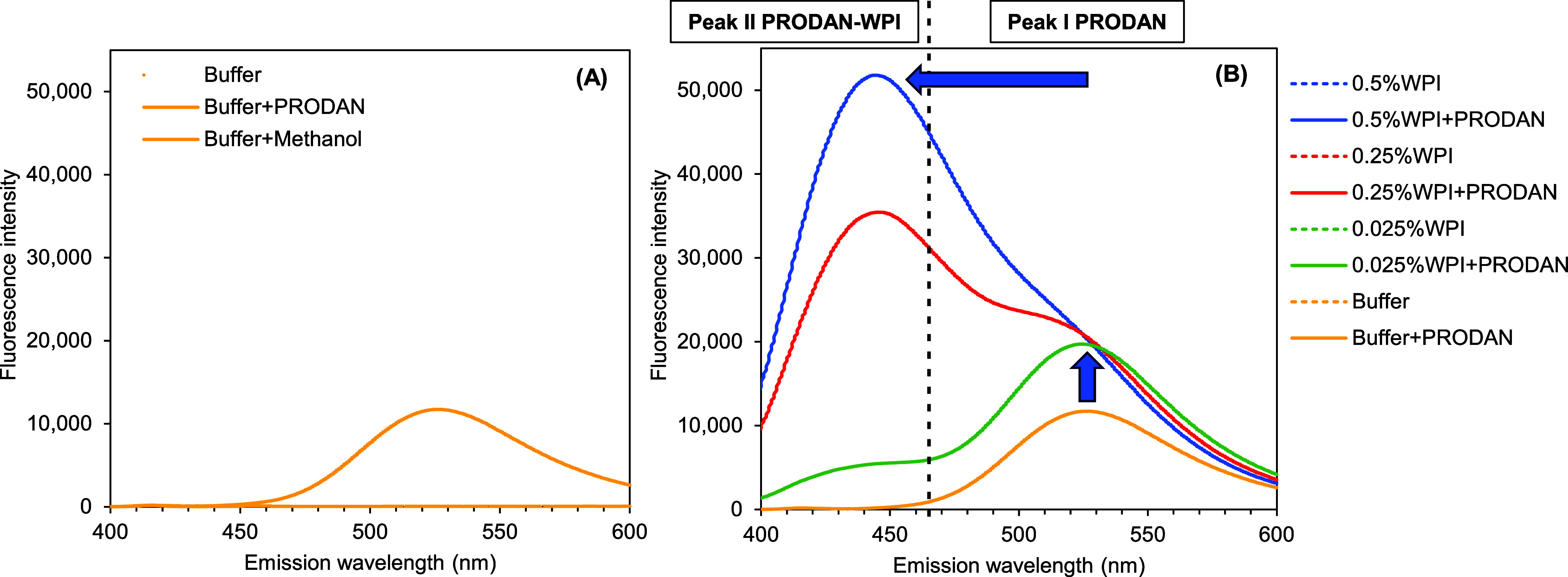
(A) Fluorescence emission spectra of McIlvaine buffer at pH 7 (dotted line); 1.41 mM PRODAN dissolved in methanol in the buffer (solid line) and methanol in the buffer (dashed line). (B) Fluorescence emission spectra of WPI control solutions (dotted lines) and 1.41 mM PRODAN in WPI solutions (solid lines) at different WPI concentrations (0%, 0.025%, 0.25%, and 0.5% (w/v) or approximately 0 mM, 0.02 mM, 0.2 mM, and 0.4 mM). The dashed vertical line indicates the RFI at a fixed wavelength of 465 nm, and the blue arrows indicate attention points discussed in the text.

The emission spectra of the McIlvaine buffer at pH 7 and the buffer with PRODAN (Figure 3A) confirm that the presence of PRODAN in the buffer solution leads to a considerable fluorescence emission at around 450 to 600 nm wavelength, with a maximum intensity at 530 nm wavelength. The addition of 0.025% (w/v) WPI increased the intensity of the original PRODAN fluorescence emission at 530 nm further (Figure 3B, peak I) but additionally resulted in a blue-shifted fluorescence emission with a second intensity peak at 445 nm wavelength (Figure 3B, peak II). This is typical for solvatochromic probes such as PRODAN:^23^ Peak I represents the PRODAN emission, while peak II represents the PRODAN-WPI emission. Chakrabarti^24^ also found that the fluorescence maximum of PRODAN shifts toward blue, which increases in intensity with decreasing solvent polarity,^25^ or in the presence of proteins with hydrophobic binding sites, or other hydrophobic binding partners.

Measuring the RFI at a fixed wavelength of 465 nm records not only a signal from the WPI-bound PRODAN (peak II) but also from the original PRODAN signal (peak I). However, a shift to a different wavelength, e.g., 430 nm, to avoid this interference is also not an option, as the blue shift of PRODAN varies depending on the environment^22^ and thus also with each binding partner. Simple subtraction of the PRODAN-buffer signal can account for that, but Figure 2 still shows a deviation from linearity at higher concentrations for PRODAN. This was probably caused by two equilibria at the same time, as follows:
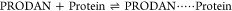
1
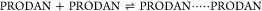
2

PRODAN exhibits self-association in water already at concentrations as low as 0.9 μM.^26^ PRODAN/PRODAN-related dipole relaxation does not occur, and therefore, PRODAN cannot quench its own fluorescence within a certain range.^25^ Furthermore, PRODAN/PRODAN association does not result in a spectral shift.^26^ Even though the emission intensity of PRODAN seems independent of the aggregation state over a large concentration range of up to 15 μM,^26^ there are indications that PRODAN self-association favors the emitting conformation. The monomeric form is more strongly affected by hydrogen-bond-induced quenching from water.^27^ This would mean that while PRODAN association (eq 2) does not cause a spectral shift, changing the equilibrium between monomeric (low emitting) and associated (high emitting) PRODAN, for example, by adding a protein or other hydrophobic binding partners, might affect the emission intensity. The subtraction of the PRODAN buffer signal from the PRODAN-protein signal can result in deviations from linearity for very high WPI: PRODAN ratios, because the second equilibrium (eq 2) will be shifted when bound to the protein.

Even though the subtraction of the RFI of WPI control solutions from those of the solutions with PRODAN and the deduction of the PRODAN fluorescence (net RFI of the buffer) are important, it is not a perfect solution to cover the complexity of the underlying interactions. It is recommended to assess the full spectrum of the PRODAN and PRODAN-protein emission when it is unclear whether or not molecules interact with the probe. When the excitation and emission wavelengths as well as the concentration ranges of WPI for ANSA and PRODAN measurements are compared, it is evident that these were different. As a result, the RFI ranges of both fluorescent probes are not comparable, meaning that the effective hydrophobicity estimates obtained from those probes cannot be quantitatively compared.

### Determination of the Effective Hydrophobicity of WPI as a Function of the pH Value

**Figure 4:**
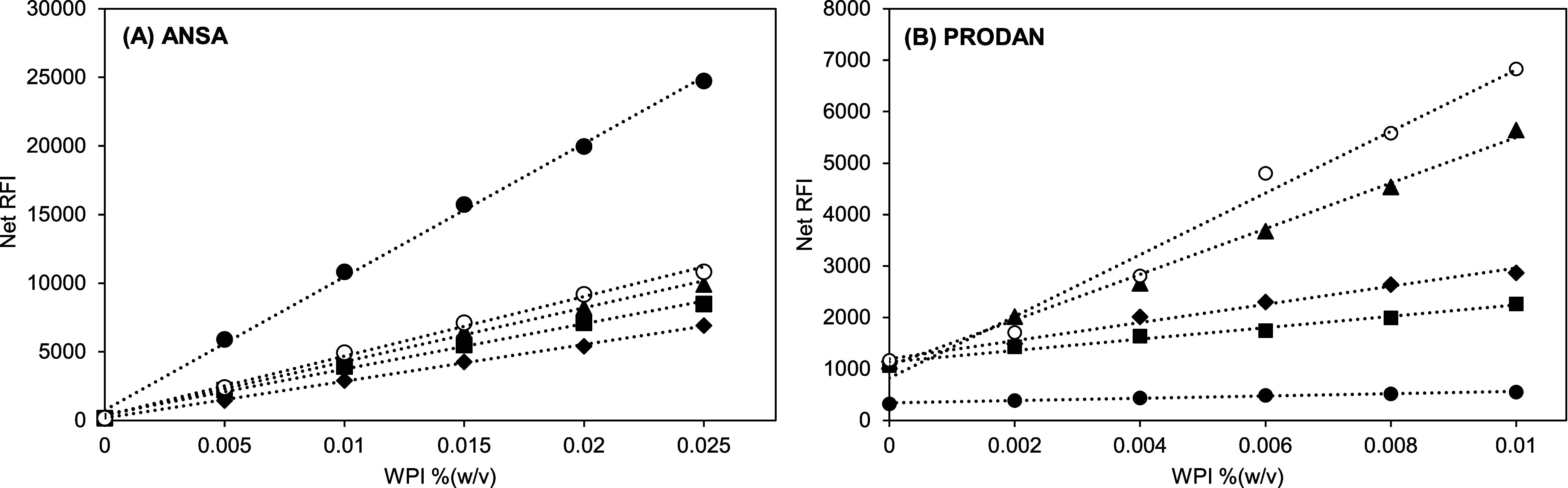
Net relative fluorescence intensity (net RFI) of WPI solutions versus the WPI concentration when using ANSA (A) and PRODAN (B) fluorescent probes at different pHs: 3 (●), 5 (■), 7 (⧫), 8 (▲), and 9 (○) (two replications; standard deviations are given, but not visible; *R*-square >98%).

**Figure 5:**
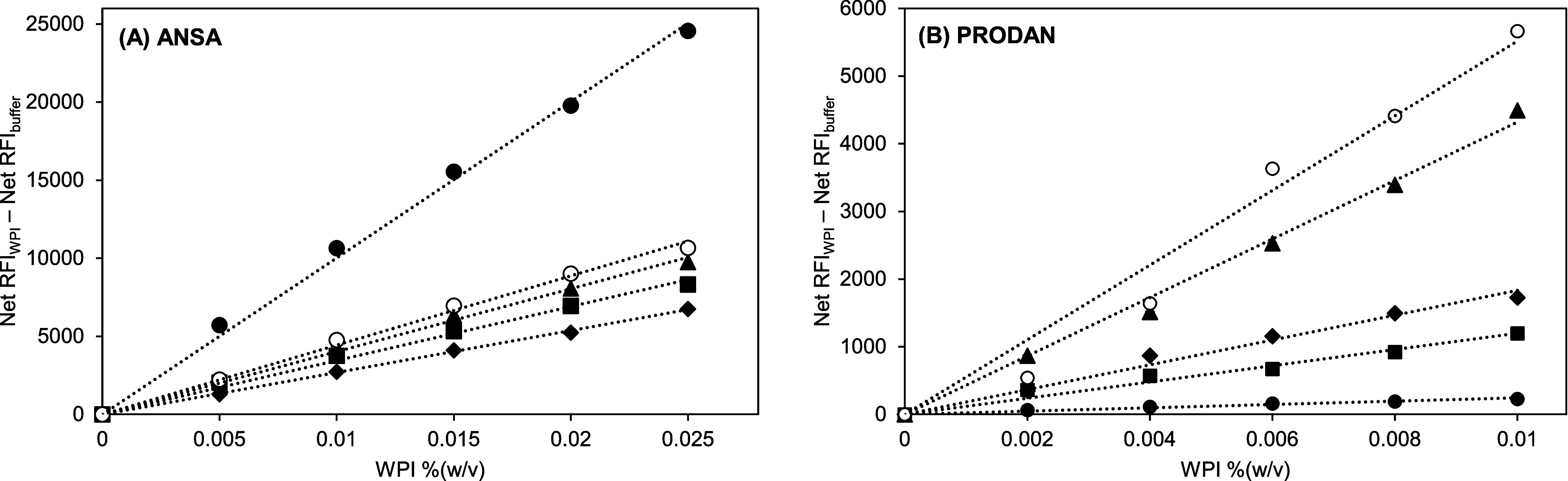
Corrected net relative fluorescence intensity (corrected net RFI*) of WPI solutions versus the WPI concentration when using ANSA (A) and PRODAN (B) fluorescent probes at different pHs: 3 (●), 5 (■), 7 (⧫), 8 (▲), and 9 (○) (two replications; standard deviations are given, but not visible; *R*-square >98%). *Corrected net RFI is the result of subtracting the net RFI of the buffer from the net RFI of WPI solution.

The net RFI estimates of WPI solutions at different pH values were plotted versus their concentrations for the ANSA and PRODAN measurements (Figure 4). The *R*-square values evaluated by linear regression for both measurements were higher than 98%, referring to a good fit for the linear model. For ANSA measurement (Figure 4A), the ordinates at *x* = 0 were small and comparable for all pHs with an average value of 174 ± 8. The presence of PRODAN in the buffer, however, shows two distinct groups of ordinates (Figure 4B). The smallest ordinate fell to 325 ± 0.6 at pH 3, while the average value was 1134 ± 42 for the other pHs. PRODAN is protonated below pH 2.75,^23^ which may reduce PRODAN aggregation under acidic conditions (repulsion), resulting in a lower RFI. Nonemitting conformational changes of PRODAN as a consequence of protonation might also be possible. We, therefore, recommend not using PRODAN at pH values below 3. Furthermore, the dissimilar ordinates at *x* = 0 for any pH and fluorescent probes imply pH-related changes in the probe–probe association. The corrected net RFIs of WPI solutions by subtracting the net RFI of the buffer from the net RFIs of WPI solutions at corresponding pH values were plotted against their concentrations and are shown in Figure 5. Linear regression was applied to the plots, and their slopes (Table S1) indicated the effective hydrophobicity and corrected effective hydrophobicity for individual pH conditions (Figure 5). The correction shows a slight difference between effective hydrophobicity and corrected effective hydrophobicity (Table S1).

**Figure 6:**
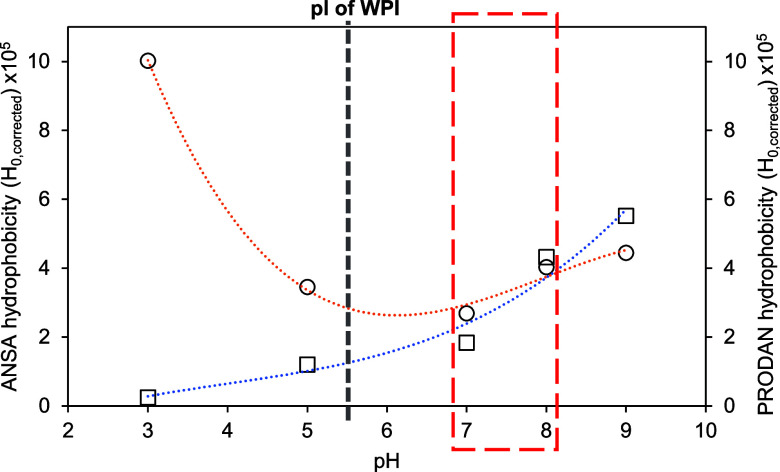
Corrected effective hydrophobicity (*H*_0,corrected_) of whey protein isolate (WPI) measured at pH 3.0–9.0 with ANSA (○) and PRODAN (□) fluorescent probes. * *H*_0,corrected_ is the slope obtained by doing linear regression (Figure 5). The red box indicates the pH at which both probes are most reliable. The gray dashed line indicates the approximate isoelectric point of the WPI proteins.

In the case of the anionic probe (Figure 6, orange dotted line), the corrected effective hydrophobicity of WPI is maximum at pH 3 at which point WPI has a net positive charge. The hydrophobicity shows a minimum at around pH 5–6 which is near the isoelectric point (pI).^16^ At higher pH values, *H*_0,corrected_ increases again. We expect that the high value at pH 3 is due to charge interaction since the probe and the protein are oppositely charged at this pH. This needs to be taken into account when using ANSA for proteins below their pI. Likewise, at the pI of WPI, the protein may have precipitated or aggregated, thus not allowing much association with any probe. At higher pH, the interaction is mostly based on hydrophobicity, although with increasing contribution of charge repulsion because the protein and the probe now have similar charges. Consequently, *H*_0,corrected_ at low pH is likely to be overestimated by ANSA because of the double effects of hydrophobic and electrostatic interactions and might be underestimated at high pH values.

With the less ionic fluorescent probe (PRODAN), the corrected effective hydrophobicity value was almost zero at pH 3 and then increased with an increase in pH (Figure 6, blue dotted line). While PRODAN is significantly less ionic (Figure 1), we may expect that the interaction is mostly based on a hydrophobic interaction. However, the tertiary amino group in PRODAN is basic as it can accept a proton. Its p*K*_b_ is likely below pH 2.75,^23^ given its naphthalene group may well be positively charged at this pH. It is, therefore, possible that the low hydrophobicity values at lower pH are influenced by the positive charge of PRODAN under these conditions. At higher pH, this charge disappears, and the hydrophobic interaction will dominate; hence, the stronger net RFI signal is observed at higher pH.

Our observations of the (corrected) effective hydrophobicity estimates of WPI at diverse pH are in agreement with the result found by Alizadeh-Pasdar and Li-Chan.^8^ They measured the surface hydrophobicity (*S*_0_) of WPI at pH 3, 5, 7, and 9 using 1-anilinonaphthalene-8-sulfonic acid (ANS) and PRODAN. Their highest *S*_0_ was obtained at pH 3 with the ANS probe, while an increase in pH increased the surface hydrophobicity of WPI when PRODAN was applied. Hence, the hydrophobicity results of both fluorescence probes can be unreliable below the pI of a protein because of attractive charges for ANS and the increasing protonation of PRODAN. For ANS, the results are also unreliable at far above the pI due to molecular repulsion. This leaves pH 7 and 8 as the most reliable for ANS and pH 7–9 for PRODAN with WPI. Still, when we compare our surface hydrophobicity results to the reported hydrophobicity and solubility of WPI under these conditions, we can find some confirming overlap: the highest solubility with 1.2 mg/mL is reported at pH 7, where a lower exposure of hydrophobic amino groups is observed, while at pH 9, only 0.75 mg/mL WPI remains soluble and the high pH starts to expose more hydrophobic amino acid residues due to unfolding.^28^

### Using the Uncharged Fluorescent Probe (PRODAN) with Protein Hydrolysate and Amino Acid

**Figure 7:**
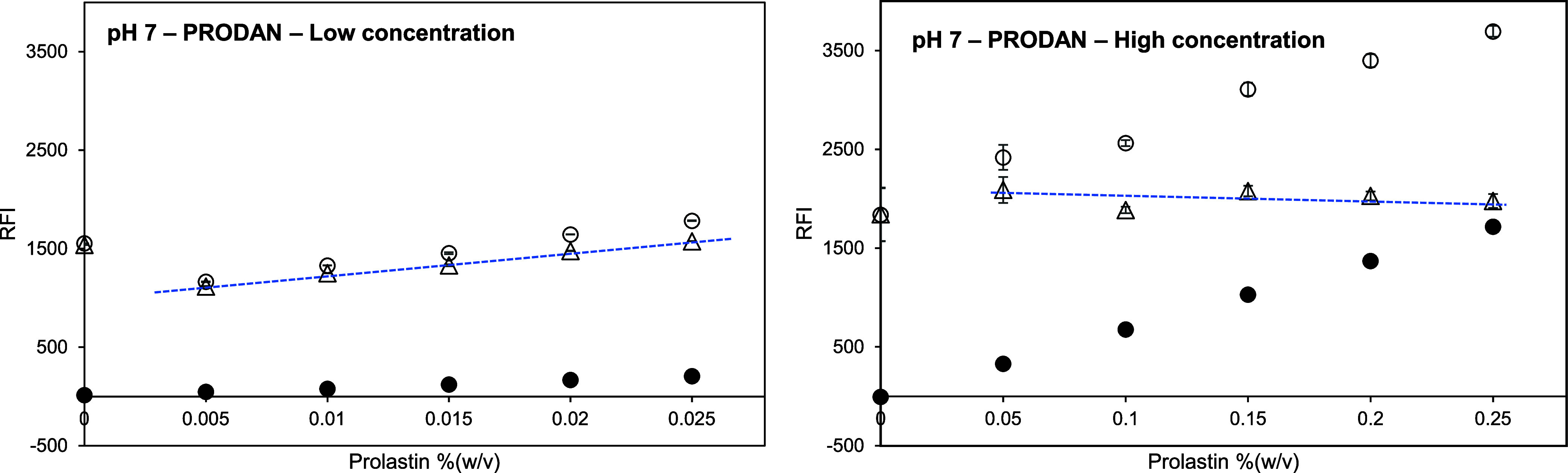
Relative fluorescence intensity (RFI) of Prolastin in McIlvaine buffer at pH 7 and 1.41 mM PRODAN in Prolastin solution at pH 7 versus Prolastin concentration (between 0.005 and 0.25%(w/v) or approximately 0.9–41.7 mM). Open symbols represent the RFI of a Prolastin solution with PRODAN. Closed symbols represent the RFI of a Prolastin control solution without PRODAN. Triangles represent the net RFI of a Prolastin solution, which is the result of subtracting the RFI of Prolastin control solution from the RFI of Prolastin solution with PRODAN. Excitation and emission wavelengths were 365 and 465 nm, respectively. The blue dotted line is for a guide for the eye.

**Figure 8:**
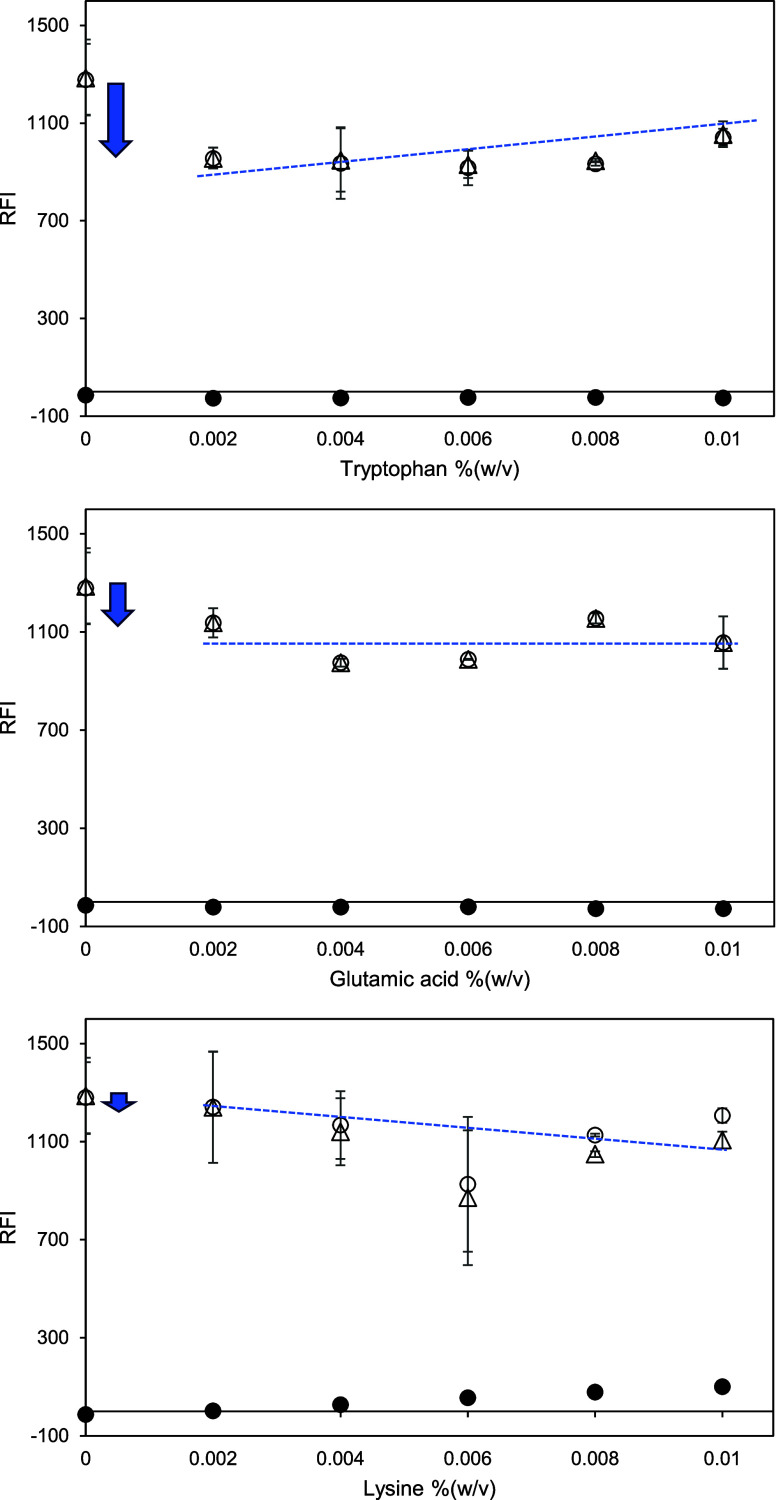
Relative fluorescence intensity (RFI) of amino acid (tryptophan = top; glutamic acid = middle; lysine = bottom) in McIlvaine buffer at pH 7 and 1.41 mM PRODAN in amino acid solution at pH 7 versus amino acid concentration (between 0.002 and 0.01% (w/v) or approximately 0.2–0.6 mM). Open symbols represent the RFI of an amino acid solution with PRODAN. Closed symbols represent the RFI of an amino acid control solution without PRODAN. Triangles represent the net RFI of an amino acid solution, which is the result of subtracting the RFI of amino acid control solution from the RFI of amino acid solution with PRODAN. Excitation and emission wavelengths were 365 and 465, respectively. The blue dotted line is for a guide for the eye, and blue arrows indicate attention points discussed in the text.

A fish protein hydrolyzate (Prolastin) with a molecular weight of 60–10,000 Da and three amino acids (Trp, Glu, Lys; average MW ∼ 165.85 Da) were assessed on their net RFI signals. The results are demonstrated in Figures 7 and 8.

Figure 7 shows the fluorescence signals of the fish protein hydrolyzate (Prolastin) solutions without and with PRODAN in different concentration ranges at pH 7. At a range of 0.005–0.025% (w/v) Prolastin (i.e., an apparent molar Prolastin: PRODAN ratio of 0.7–3.0), the RFI of control and Prolastin solutions and their net intensity increased with increasing concentration. However, the RFI and net RFI values of Prolastin solutions were lower than those of the buffer (at 0% (w/v) Prolastin) at all concentrations. This suggests that the presence of hydrolyzate quenched the probe RFI. To further study this effect, the concentration range of Prolastin was increased to 0.05–0.25% (w/v) (i.e., an apparent molar Prolastin: PRODAN ratio of 6.0–29.6). The results are depicted on the right part of Figure 7. Now, the RFI values of Prolastin solutions with PRODAN were higher than the net RFI of the buffer, and the net RFIs of Prolastin solutions were comparable to the value of the buffer. This indicates that the PRODAN measurement did not work for determining the effective hydrophobicity of our fish protein hydrolyzate. Similar results were found at the other pH conditions (pH 3, 8, 5, and 9) for both concentration ranges, as seen in Figures S5–S6. A similar quenching effect was observed when individual amino acids were added instead of Prolastin (Figure 8).

Tryptophan is a hydrophobic amino acid, solubility in water at 25 °C: 1.14%(w/v),^29^ while glutamic acid and lysine are hydrophilic amino acids with electrically charged side chains at pH 7. Glutamic acid and lysine are the majority of the charged amino acids in Prolastin.^30^ Tryptophan and glutamic acid provided comparable results with the PRODAN measurement (Figure 8, top and middle). The amino acids slightly absorbed the signal at the emission wavelength, resulting in small negative RFI values of the amino acid control solutions at all concentrations. In addition, the intensity values of adding PRODAN to amino acid solutions were equal to those after calculating the net RFIs of the solutions at each concentration. The net RFI estimate of McIlvaine buffer at pH 7 (at 0%) was greater than all intensity numbers from the solutions containing those amino acid molecules. This is consistent with findings that tryptophan binding (through pi-pi stacking and hydrophobic interactions) to PRODAN may quench the RFI of PRODAN by electron transfer (photoreduction).^31^ The quenching thus does indicate an interaction between the amino acids and PRODAN. This is not limited to free tryptophan but includes the tryptophan in proteins and peptides. Since quenching is unaffected by the increasing tryptophan concentration, PRODAN-PRODAN and perhaps tryptophan-tryptophan^32^ self-association is more favorable than PRODAN-tryptophan interaction.

It is unclear whether glutamic acid also quenches PRODAN through static binding, because the observed RFI decrease was relatively low (Figure 8, middle). For PRODAN, hydrogen bonding with its negatively charged carbonyl oxygen has been reported, and these bonds are stronger in the excited state than in the ground state.^33^ However, the glutamic acid carbonyl group is negatively charged at pH 7, and thus, interactions would be more likely with lysine. The observation that the RFI values of the lysine control solutions were not negative does not imply that lysine absorbs the signal during the measurement, as the RFI and net RFI values of lysine solutions are still smaller than the net intensity of the buffer. A higher concentration of lysine did not produce a higher fluorescence intensity. This suggests that PRODAN molecules do bind to tryptophan but not to glutamic acid or lysine. Perhaps the addition of these two amino acids affected the PRODAN self-association and PRODAN-water hydrogen bonds, thereby indirectly changing the equilibrium between low-emissivity monomers and high-emissivity aggregates of PRODAN.

**Figure 9:**
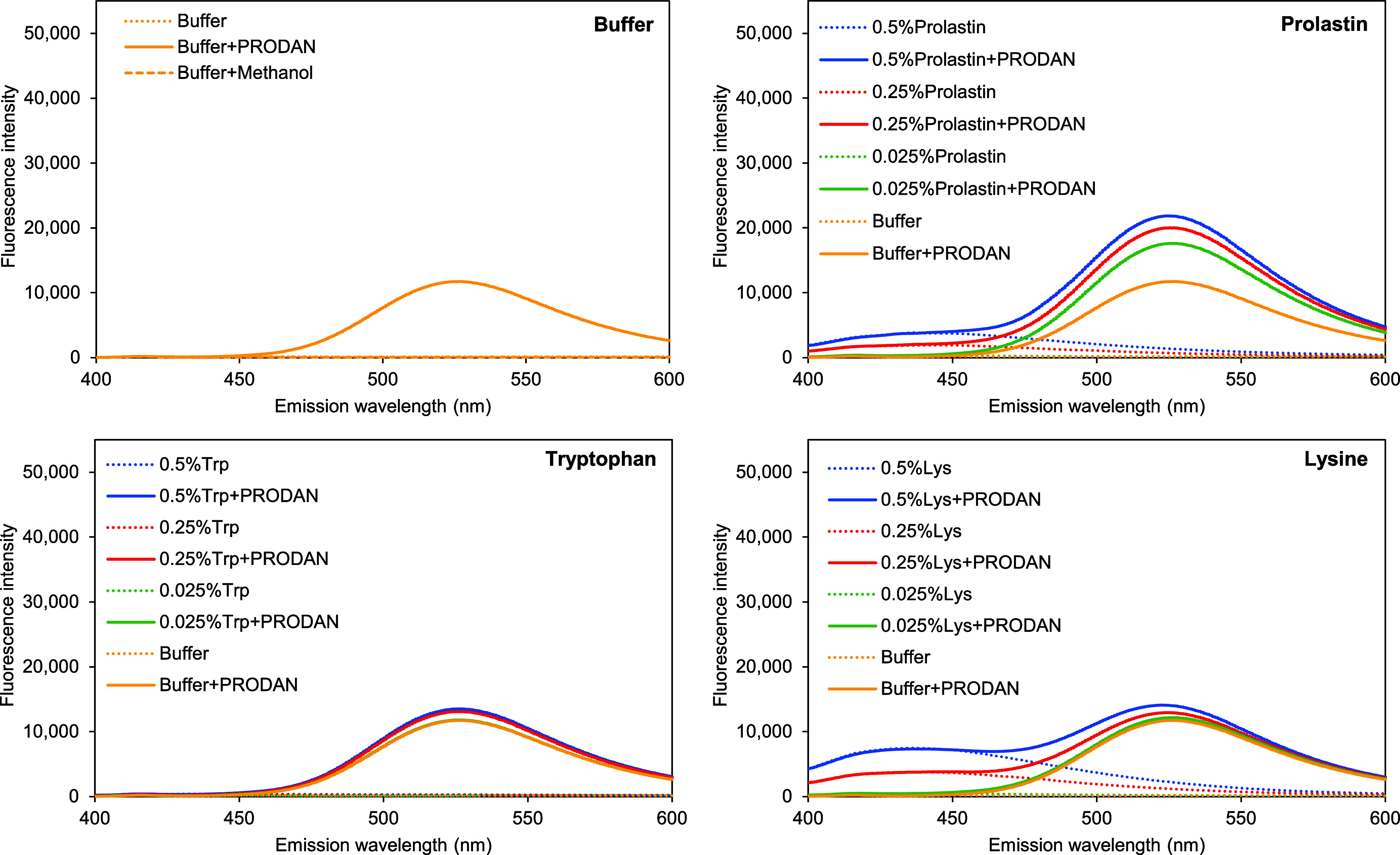
Fluorescence emission spectra of protein control solutions (Prolastin/tryptophan/lysine) and 1.41 mM PRODAN in McIlvaine buffer at pH 7 and in Prolastin/Tryptophan/Lysine solutions at different concentrations (0%, 0.025%, 0.25%, and 0.5% (w/v) or approximately 0 mM, 4.2 mM, 41.7 mM, and 83.3 mM for Prolastin and 0 mM, 1.5 mM, 15.1 mM, and 30.1 mM for tryptophan/lysine).

The observed comparable quenching effects of tryptophan and Prolastin indicate that the fish protein hydrolyzate may be rich in accessible aromatic amino acids such as tryptophan, tyrosine, phenylalanine, and proline. Hydrophobic interactions and pi-stacking with PRODAN would result in electron transfer-based static quenching of the fluorescence probe, reducing the observed RFI instead of blue-shifting it (Figure 9). This effect is not observed in an excess of tryptophan or Prolastin, which also indicates that PRODAN-PRODAN and probably Prolastin-Prolastin self-associations are much stronger than the PRODAN-Prolastin association. The emission spectra also show that measuring the RFI at a fixed wavelength can be misleading because Prolastin and lysine show a strong intrinsic fluorescence signal at 430 nm, which could have been misinterpreted as a PRODAN blueshift. Overall, due to the complex interactions, PRODAN is not a suitable probe to assess individual amino acids or peptides with a high amount of solvent-accessible aromatic amino acids, as quenching effects occur instead of a blue shift.

The emission spectra of protein solutions with PRODAN (Figure 9) were measured in the presence of excess Prolastin or amino acid (up to 30 mM amino acid per 1.41 mM PRODAN). Unlike with WPI (Figure 3), no blueshift was observed for all components at any concentration. The emission spectra of tryptophan looked identical to the spectrum of the buffer. An increase in tryptophan concentration did not increase the fluorescence intensity. The same observation was found for the spectra of glutamic acid (Figure S10). The spectra of tryptophan confirm the assumption that tryptophan addition already reached a maximum binding to PRODAN at 0.1 mM or 0.002% (w/v) (Figure 8), with further excess having no further effect on the PRODAN binding or equilibrium. It also confirms that PRODAN-tryptophan binding does not result in a spectral shift.

In the cases of Prolastin and lysine, we found that the presence of the protein hydrolyzate molecules and positively charged solutes produced fluorescence over emission wavelengths between 400 and 550 nm. Larger Prolastin and lysine concentrations enhanced this intensity. This was also observed in several other studies.^34−36^ For instance, Sahadevan et al.^36^ reported an increase in fluorescence intensity over the emission range of 350–650 nm wavelength (excitation at 365 nm) with increasing lysine concentration of up to 100 mM. This intrinsic emission from nonaromatic amino acids originates from their aggregation^34,35^ via ‘clustering-triggered emission.^35^ Adding PRODAN to the protein solutions did not provide any blueshift over the emission wavelength.

We showed that adding PRODAN to solutions containing small molecules such as peptides and amino acids does not provide RFI values from the hydrophobic interaction between PRODAN molecules and the hydrophobic areas of the small molecules. The uncharged fluorescent probe thus cannot be used to evaluate the effective hydrophobicity of the amino acids and fish protein hydrolyzate in this study. This highlights that considering all values from measuring fluorescence intensity is important before interpreting the results as effective hydrophobicity, particularly in the case of using PRODAN with small molecules.

### Using the Anionic Fluorescent Probe (ANSA) with Protein Hydrolyzate and Amino Acid

**Figure 10:**
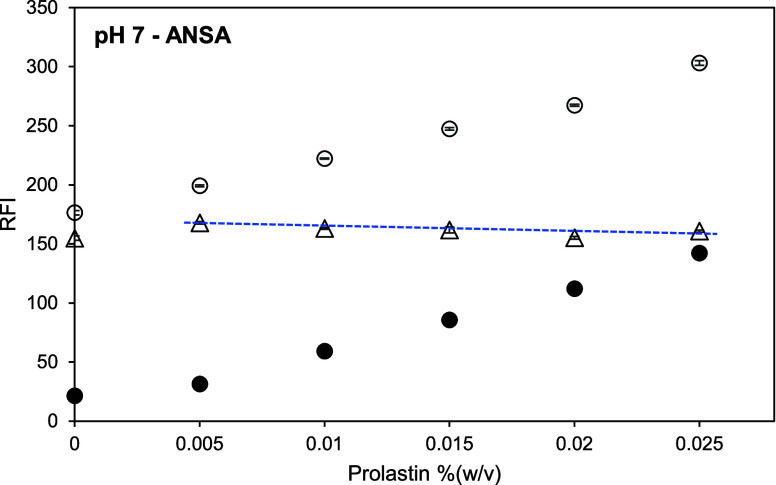
Relative fluorescence intensity (RFI) of Prolastin in McIlvaine buffer at pH 7 and 8 mM ANSA in Prolastin solution at pH 7 versus Prolastin concentration (between 0.005 and 0.025% (w/v) or approximately 0.9–4.2 mM). Open symbols represent the RFI of a Prolastin solution with ANSA. Closed symbols represent the RFI of a Prolastin control solution without ANSA. Triangles represent the net RFI of a Prolastin solution, which is the result of subtracting the RFI of Prolastin control solution from the RFI of Prolastin solution with ANSA. Excitation and emission wavelengths were 390 and 470 nm, respectively. The blue dotted line is for a guide for the eye.

**Figure 11:**
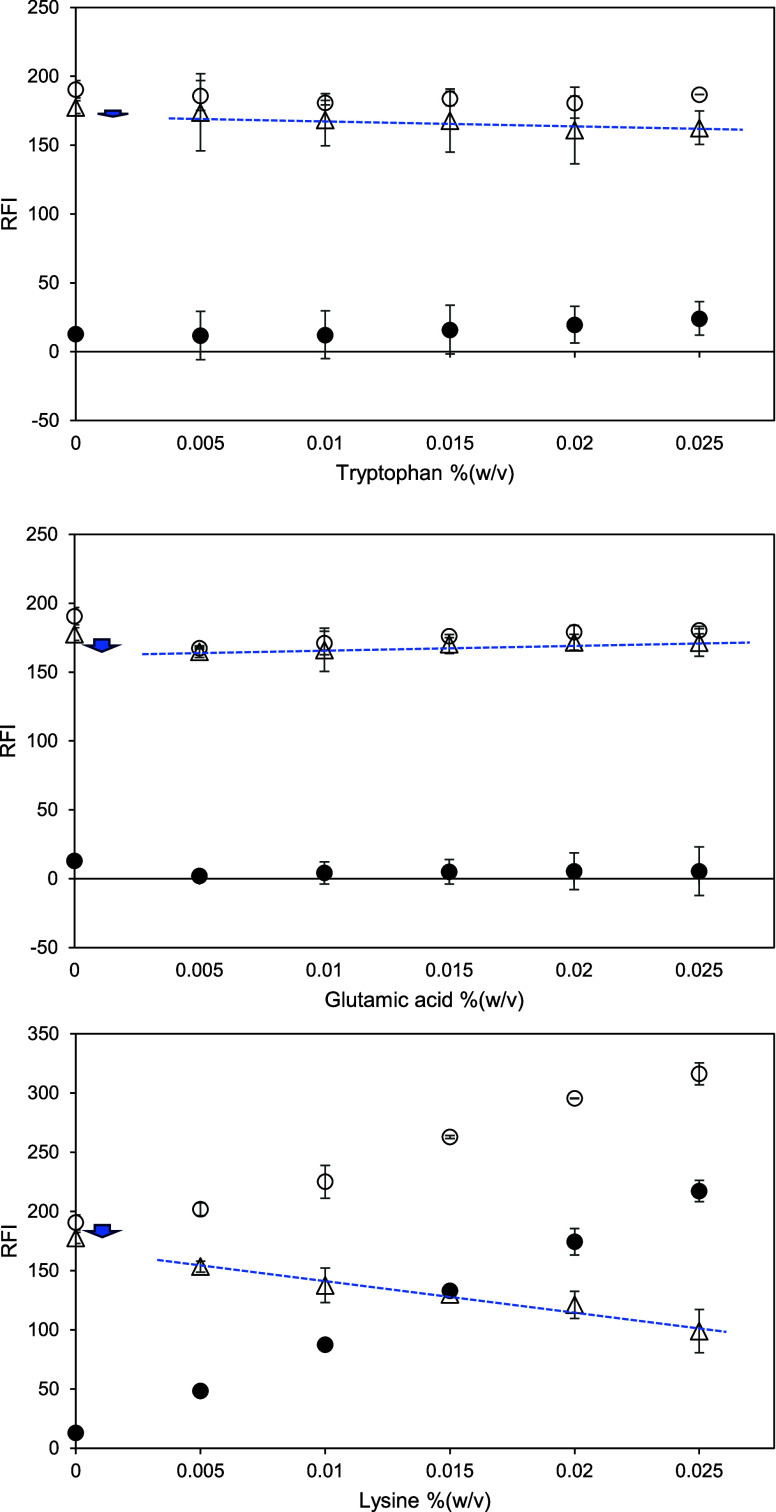
Relative fluorescence intensity (RFI) of amino acid (tryptophan = top; glutamic acid = middle; lysine = bottom) in McIlvaine buffer at pH 7 and 8 mM ANSA in amino acid solution at pH 7 versus amino acid concentration (between 0.005 and 0.025% (w/v) or approximately 0.3–1.5 mM). Open symbols represent the RFI of an amino acid solution with ANSA. Closed symbols represent the RFI of an amino acid control solution without ANSA. Triangles represent the net RFI of an amino acid solution, which is the result of subtracting the RFI of amino acid control solution from the RFI of amino acid solution with ANSA. Excitation and emission wavelengths were 390 and 470 nm, respectively. The blue dotted line is for a guide for the eye, and blue arrows indicate attention points discussed in the text.

Next using PRODAN, also ANSA was used with Prolastin, and the three amino acids were done using the charged fluorescent probe (ANSA). All results from ANSA measurements are shown in Figures 10–11 and S11 .

Even though the net RFI of ANSA in the buffer (at 0% (w/v)) was not completely zero (Figures 10–11), its value was not as high as the signal from PRODAN in the buffer (Figures 7–8). This is because self-association of ANSA in polar solvents like water is low.^18,19,37^ Adding other binding candidates such as proteins, does not enhance the emission intensity of the ANSA self-interaction, and ANSA-bound protein creates a blueshift of just one peak over an emission range of 425–650 nm wavelength.^38−40^ This means that the fluorescence emission intensity is almost completely visible from the binding between ANSA and the protein. However, nonaromatic amino acids and their polymers can produce intrinsic emission^34−36^ by the clustering-triggered emission mechanism.^35^ Therefore, deduction of the RFI values of the control solutions is still important.

The RFI signals rose with an increasing Prolastin concentration for Prolastin solutions with ANSA and Prolastin control solutions (without ANSA). However, the net RFIs by subtracting the RFI of control solutions from the intensity of Prolastin solutions with ANSA at corresponding concentrations do not give a similar trend with higher Prolastin concentrations. The net RFI values of Prolastin solutions were the equivalent of the net RFI of ANSA in McIlvaine buffer at pH 7 (the net RFI at 0% (w/v) Prolastin) and gradually decreased with increasing hydrolyzate concentration. The reduction of the net fluorescence intensity could be due to Prolastin aggregation. Aggregated peptides generate their own intrinsic emission but may also impede the self-association of ANSA. Another explanation is a quenching of tryptophan residues from increasing Prolastin concentration, enhancing the association of Prolastin molecules. This leads to molecular aggregation, reducing the accessibility of ANSA. This is in line with the study of Sironi et al.,^41^ who found decreasing fluorescence intensity at higher concentrations of gluten, which is explained by gluten association that eliminates the exposure of hydrophobic sites.

The anionic fluorescent probe (ANSA) was used with the same three amino acids. The absolute and net RFI values are plotted in Figure 11. The fluorescence signals obtained from the control solutions of the hydrophobic and negatively charged amino acids were close to zero. The RFIs of the tryptophan control solutions slowly increased with concentration (Figure 11, top), possibly from tryptophan-tryptophan interaction, given its intrinsic emission.^32^ Addition of ANSA to the buffer solution gives a higher RFI of up to about 190, due to self-association of ANSA, as previously discussed. Increasing the tryptophan concentration does not yield a higher fluorescence. The intensity with ANSA slightly dropped when adding tryptophan. This implies that tryptophan hardly quenches the fluorescence of ANSA and does not bind to ANSA. This is supported by Prajapati et al.^32^ Their spectra of ANS in phosphate buffer and in ANS-tryptophan mixture were comparable over an emission wavelength of 450–600 nm.

For glutamic acid (Figure 11, middle), no intrinsic fluorescence was observed. Here is no indication of self-association, as glutamic acid is negatively charged at pH 7. The presence of glutamic acid did not decrease the fluorescence intensity of ANSA in any significant way. This means that there are no associations between amino acids or with ANSA. Lysine (Figure 11, bottom) showed the increased fluorescence of the control solutions, through clustering-triggered emission.^34−36^ The fluorescence of lysine solutions with ANSA was dependent on its concentration, probably by self-association of the positively charged amino acids. On the other hand, the net florescence of lysine solutions gradually decreased at higher amino acid concentrations. The net fluorescence of lysine solutions with ANSA was also lower than the net value at 0% (w/v) lysine. This means that the ANSA probe does not associate with lysine, but interestingly also that lysine does interfere with the probe-probe interaction. The above results clearly reveal that the anionic fluorescent probe (ANSA) cannot be applied to protein hydrolyzates (such as Prolastin) and the amino acids in this study. This is because any net relative fluorescence does not originate from the interaction between the probe and the hydrophobic regions of the components but from other interactions.

Fluorescent probe methods are commonly used to assess the effective/surface hydrophobicity of proteins to study their structures and properties in changing environments.^8,9,16,42−45^ These probes are also employed to measure the effective hydrophobicity of protein hydrolyzates, which may be related to the changed functionalities of protein hydrolyzates after processing, especially hydrolysis and heating.^46−56^ Most researchers apply a similar protocol to measure the RFI with a fluorescent probe and determine the effective/surface hydrophobicity using the net RFI values versus the concentration.^16,42,44,47−50,53,57^ Some studies did not subtract the fluorescence of control solutions,^43,46,51,52,54−56^ which results in unreliable results, since our study showed that the intensity may not (only) be produced by the hydrophobic interaction of the target components with the probe. Therefore, it is important that the type of association that is responsible for the fluorescence values be identified before applying linear regression analysis to obtain *H*_0_. This is particularly so in the case of protein hydrolyzates containing small molecules.

In conclusion, the anionic (ANSA) and uncharged (PRODAN) fluorescent probes were assessed for the determination of the effective hydrophobicity of whey protein isolate and small molecules (Prolastin, tryptophan, glutamic acid, and lysine). For WPI, both fluorescent probes can be used reliably to estimate the protein hydrophobicity. However, as ANSA is an anionic fluorescent probe, electrostatic interaction may interfere, which may lead to an overestimation of hydrophobicity. To prevent this, an anionic probe should not be used under acidic conditions, and protein hydrophobicity obtained at different pH values using ANSA cannot be compared. Furthermore, protein hydrophobicity estimations from different probes are not quantitatively comparable because of the differences in excitation and emission wavelengths and concentration ranges. For both proteins and protein hydrolyzates, the source of all fluorescence has to be identified first to be confident that the obtained fluorescence intensity is indeed caused by the interaction of the probe and the hydrophobic regions of the targeted molecules. ANSA and PRODAN cannot be applied to evaluate the effective hydrophobicity of small molecules (Prolastin, tryptophan, glutamic acid, and lysine; MW ∼ 60–10,000 Da) as there is no indication that any association takes place. In addition, it is interesting to study other types of protein hydrolyzates to narrow down the molecular size ranges in which this method can provide reliable results. Advanced technologies, such as chromatography, could be used for validating the fluorescence probe method for future research.

## Supporting Information Available

Relative fluorescence intensity vs concentration for whey protein isolate and Prolastin at other pHs (3, 5, 8, and 9); raw fluorescence emission spectra of McIlvaine buffer, whey protein isolate, Prolastin, tryptophan, lysine, and glutamic acid; effective hydrophobicity (*H*_0_) and corrected effective hydrophobicity (*H*_0,corrected_) of whey protein isolate (PDF)

## Abbreviations

ANSA: 8-anilino-1-naphthalenesulfonic acid ammonium salt
ANS: 8-anilinonaphthalene-1-sulfonic acid
PRODAN: 6-propionyl-2-(N,N-dimethylamino) naphthalene
MW: molecular weight
rp: tryptophan
Glu: glutamic acid
Lys: lysine
RFI: relative fluorescence intensity
WPI: whey protein isolate
BSA: bovine serum albumin
OVA: ovalbumin
*H*_0_: effective hydrophobicity
*S*_0_: surface hydrophobicity
pI: isoelectric point
