# Supplementary material for: Evaluating and Validating the Fluorescent Probe Methodology for Measuring the Effective Hydrophobicity of Protein, Protein Hydrolyzate, and Amino Acid
Source: J Agric Food Chem. 2024 Nov 30;72(49):27429–39. doi: 10.1021/acs.jafc.4c07664 (PMC11638966; doi:10.1021/acs.jafc.4c07664)
Supplement: Supplementary file 1 — jf4c07664_si_001.pdf [file jf4c07664_si_001.pdf]

Supporting Information

Evaluating and validating the fluorescent probe methodology measuring the effective hydrophobicity of protein, protein hydrolysate and amino acid

Nattawan Chorhirankul[a]*, Anja E.M. Janssen[a], Remko M. Boom[a] and Julia K. Keppler[a]

[a] *Food Process Engineering Group, Wageningen University, P.O. Box 17 Wageningen 6700 AA, The Netherlands*

* Email: nattawan.chorhirankul@wur.nl

Contents

Figure S1. Relative fluorescence intensity (RFI) of whey protein isolate (WPI) in McIlvaine buffer and 8 mM ANSA in WPI solution versus WPI concentration at pH 3, 5, 8 and 9.

Figure S2. Relative fluorescence intensity (RFI) of whey protein isolate (WPI) in McIlvaine buffer and 1.41 mM PRODAN in WPI solution versus WPI concentration at pH 3, 5, 8 and 9.

Figure S3. Fluorescence emission spectra of McIlvaine buffer at pH 7 (Buffer); 1.41 mM PRODAN dissolved in methanol in the buffer (Buffer+P) and methanol in the buffer (Buffer+M).

Figure S4. Fluorescence emission spectra of WPI control solutions at 0.025% (0.025), 0.25% (0.25) and 0.5% (0.5) (w/v) and 1.41 mM PRODAN in 0.025% (0.025P), 0.25% (0.25P) and 0.5% (0.5P) (w/v) WPI solutions.

Table S1. Effective hydrophobicity (H_0) and corrected effective hydrophobicity ($H_{0,corrected}$) of whey protein isolate.

Figure S5. Relative fluorescence intensity (RFI) of Prolastin in McIlvaine buffer and 1.41 mM PRODAN in Prolastin solution versus Prolastin concentration at pH 3, 5, 8 and 9; low concentrations.

Figure S6. Relative fluorescence intensity (RFI) of Prolastin in McIlvaine buffer and 1.41 mM PRODAN in Prolastin solution versus Prolastin concentration at pH 3, 5, 8 and 9; high concentrations.

Figure S7. Fluorescence emission spectra of Prolastin control solutions at 0.025% (0.025), 0.25% (0.25) and 0.5% (0.5) (w/v) and 1.41 mM PRODAN in 0.025% (0.025P), 0.25% (0.25P) and 0.5% (0.5P) (w/v) Prolastin solutions.

Figure S8. Fluorescence emission spectra of tryptophan control solutions at 0.025% (0.025), 0.25% (0.25) and 0.5% (0.5) (w/v) and 1.41 mM PRODAN in 0.025% (0.025P), 0.25% (0.25P) and 0.5% (0.5P) (w/v) tryptophan solutions.

Figure S9. Fluorescence emission spectra of lysine control solutions at 0.025% (0.025), 0.25% (0.25) and 0.5% (0.5) (w/v) and 1.41 mM PRODAN in 0.025% (0.025P), 0.25% (0.25P) and 0.5% (0.5P) (w/v) lysine solutions.

Figure S10. Fluorescence emission spectra of glutamic acid control solutions at 0.025% (0.025), 0.25% (0.25) and 0.5% (0.5) (w/v) and 1.41 mM PRODAN in 0.025% (0.025P), 0.25% (0.25P) and 0.5% (0.5P) (w/v) glutamic acid solutions.

Figure S11. Relative fluorescence intensity (RFI) of Prolastin in McIlvaine buffer and 8 mM ANSA in Prolastin solution versus Prolastin concentration at pH 3, 5, 8 and 9.

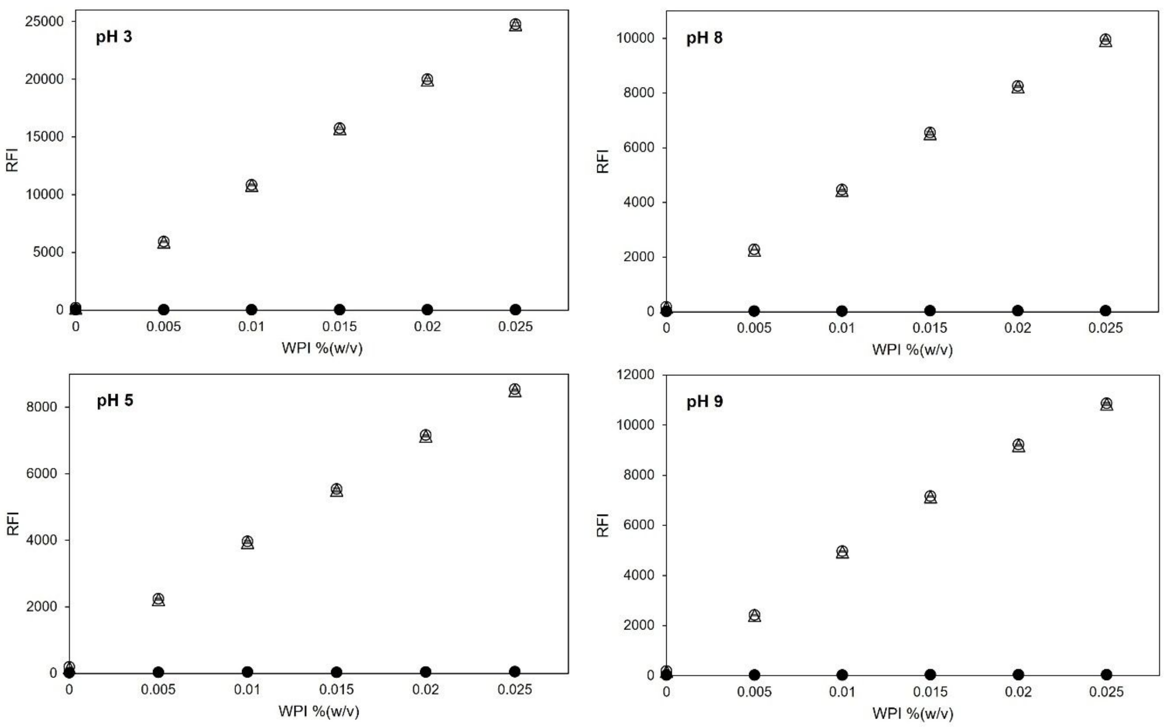

Figure S1. Relative fluorescence intensity (RFI) of whey protein isolate (WPI) in McIlvaine buffer and 8 mM ANSA in WPI solution versus WPI concentration. Open symbols (○) represent the RFI of a WPI solution with ANSA. Closed symbols (●) represent the RFI of a WPI control solution without ANSA. Triangles (Δ) represent the net RFI of a WPI solution, which is the result of subtracting the RFI of WPI control solution from the RFI of WPI solution with ANSA. Excitation and emission wavelengths for ANSA were 390 and 470 nm, respectively.

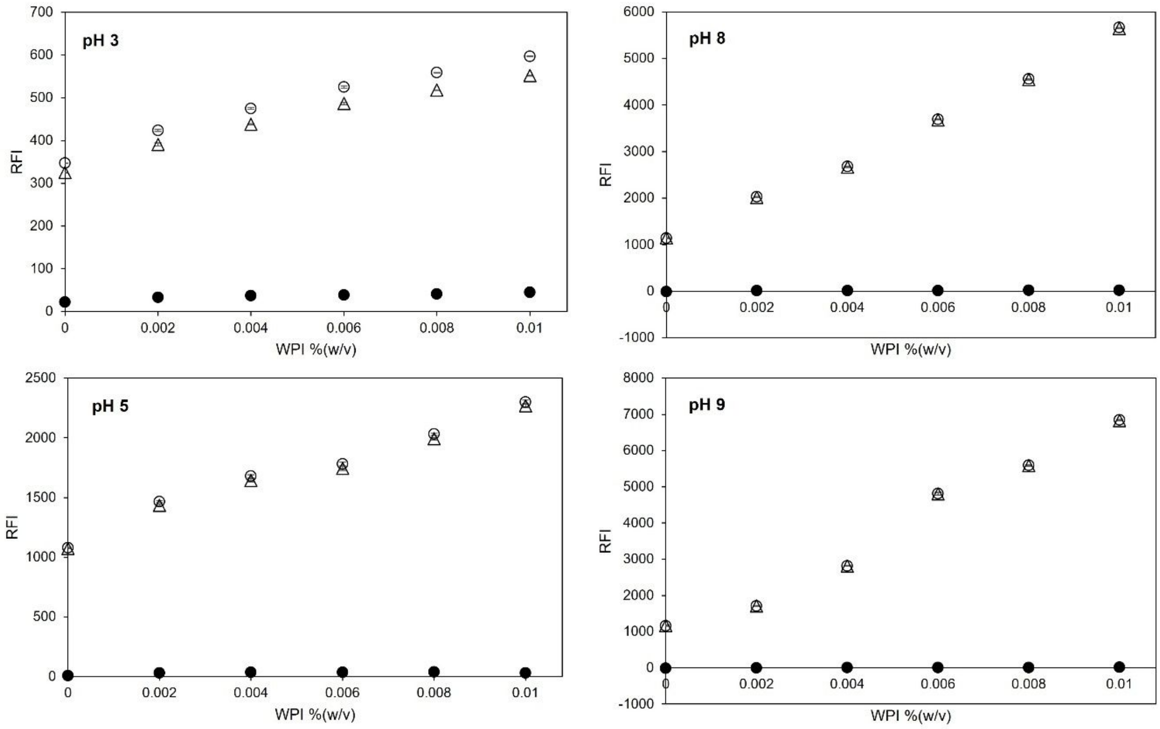

Figure S2. Relative fluorescence intensity (RFI) of whey protein isolate (WPI) in McIlvaine buffer and 1.41 mM PRODAN in WPI solution versus WPI concentration. Open symbols (○) represent the RFI of a WPI solution with PRODAN. Closed symbols (●) represent the RFI of a WPI control solution without PRODAN. Triangles (Δ) represent the net RFI of a WPI solution, which is the result of subtracting the RFI of WPI control solution from the RFI of WPI solution with PRODAN. Excitation and emission wavelengths for PRODAN were 365 and 465 nm, respectively.

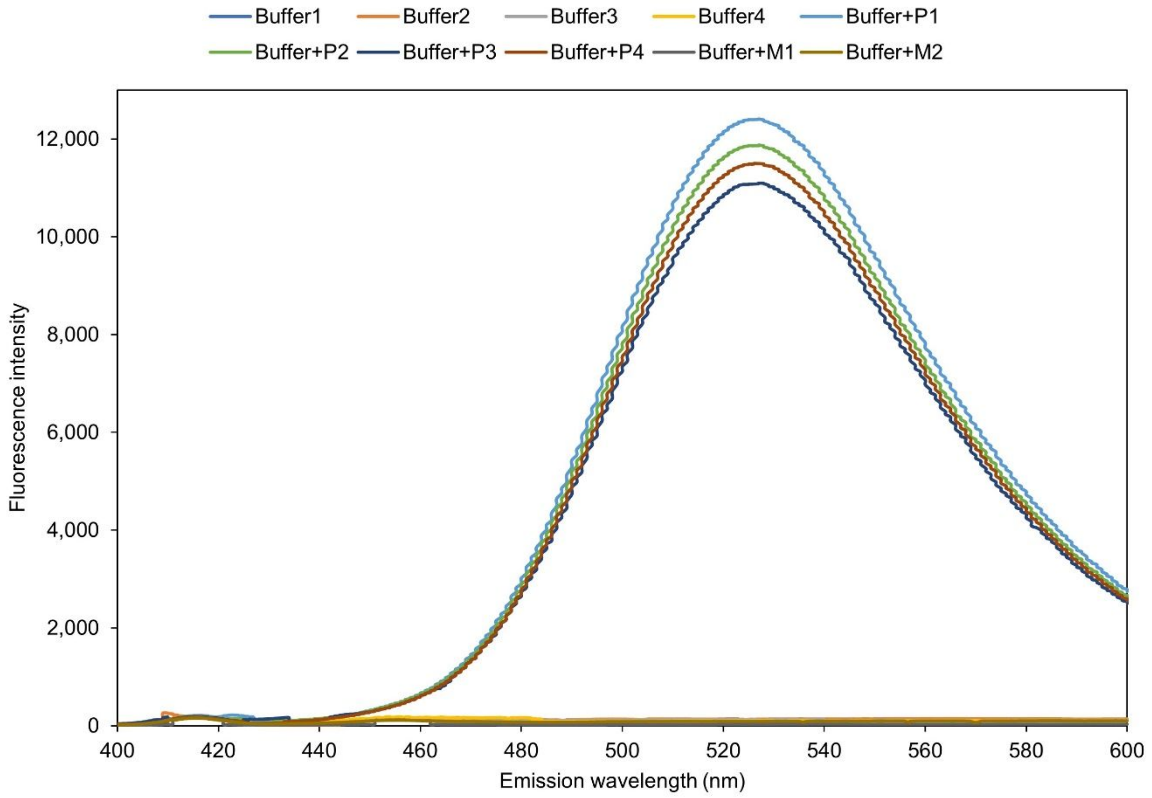

Figure S3. Fluorescence emission spectra of McIlvaine buffer at pH 7 (Buffer); 1.41 mM PRODAN dissolved in methanol in the buffer (Buffer+P) and methanol in the buffer (Buffer+M).

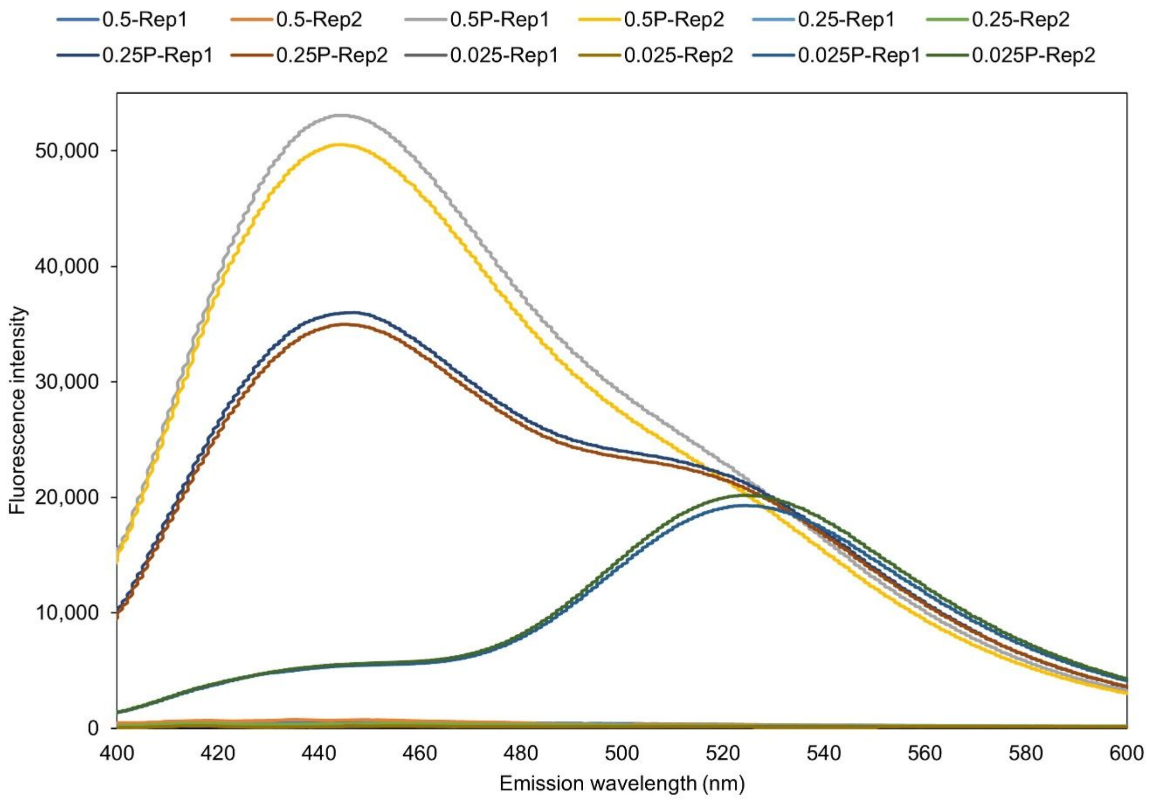

Figure S4. Fluorescence emission spectra of WPI control solutions at 0.025% (0.025), 0.25% (0.25) and 0.5% (0.5) (w/v) and 1.41 mM PRODAN in 0.025% (0.025P), 0.25% (0.25P) and 0.5% (0.5P) (w/v) WPI solutions.

Table S1. Effective hydrophobicity (H_0) and corrected effective hydrophobicity ($H_{0,corrected}$) of whey protein isolate.

**pH**	**Effective hydrophobicity (H_0 x 10^5)**		**Corrected effective hydrophobicity ($H_{0,corrected}$ x 10^5)**	
	**ANSA**	**PRODAN**	**ANSA**	**PRODAN**
**3**	9.7	0.2	10.0	0.2
**5**	3.3	1.1	3.5	1.2
**7**	2.7	1.8	2.7	1.8
**8**	3.9	4.4	4.0	4.3
**9**	4.3	6.0	4.4	5.5

Note: H_0 is the slope obtained by doing linear regression between net relative fluorescence intensity of WPI solutions versus the WPI concentration (Figure 4). $H_{0,corrected}$ is the slope obtained by doing linear regression between net relative fluorescence intensity of WPI solutions subtracted by the net relative fluorescence intensity of buffer versus the WPI concentration (Figure 5).

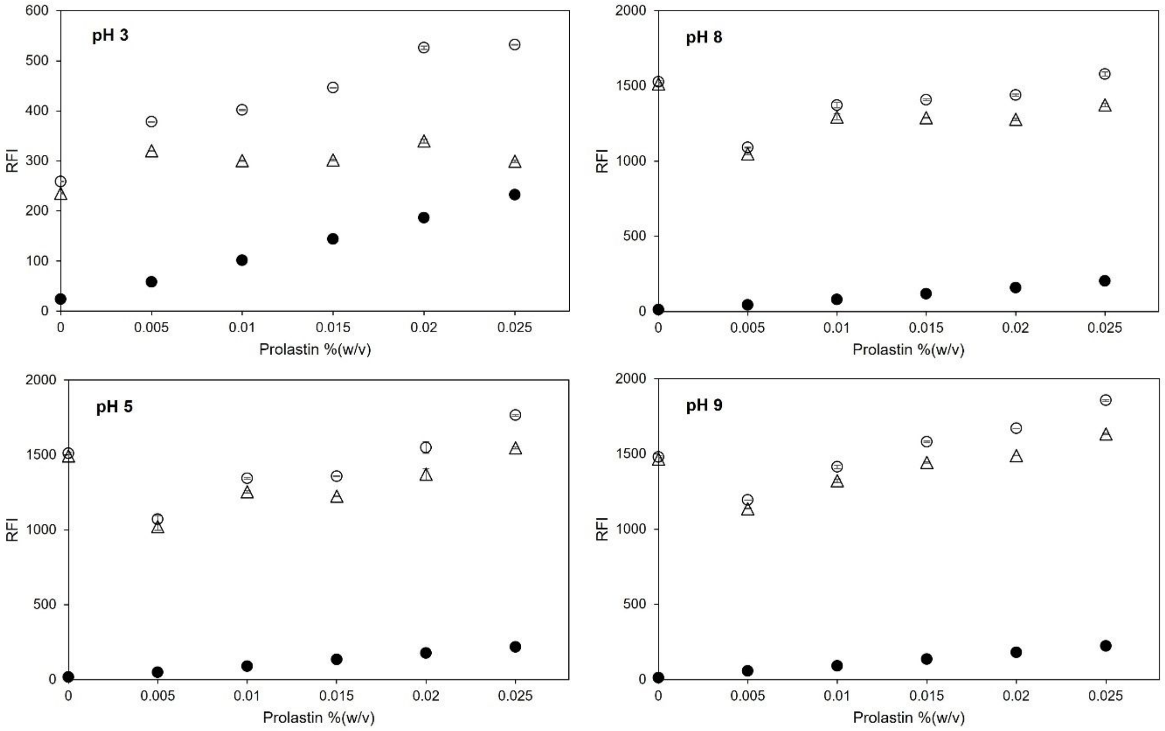

Figure S5. Relative fluorescence intensity (RFI) of Prolastin in McIlvaine buffer and 1.41 mM PRODAN in Prolastin solution versus Prolastin concentration. Open symbols (○) represent the RFI of a Prolastin solution with PRODAN. Closed symbols (●) represent the RFI of a Prolastin control solution without PRODAN. Triangles (Δ) represent the net RFI of a Prolastin solution, which is the result of subtracting the RFI of Prolastin control solution from the RFI of Prolastin solution with PRODAN. Excitation and emission wavelengths for PRODAN were 365 and 465 nm, respectively.

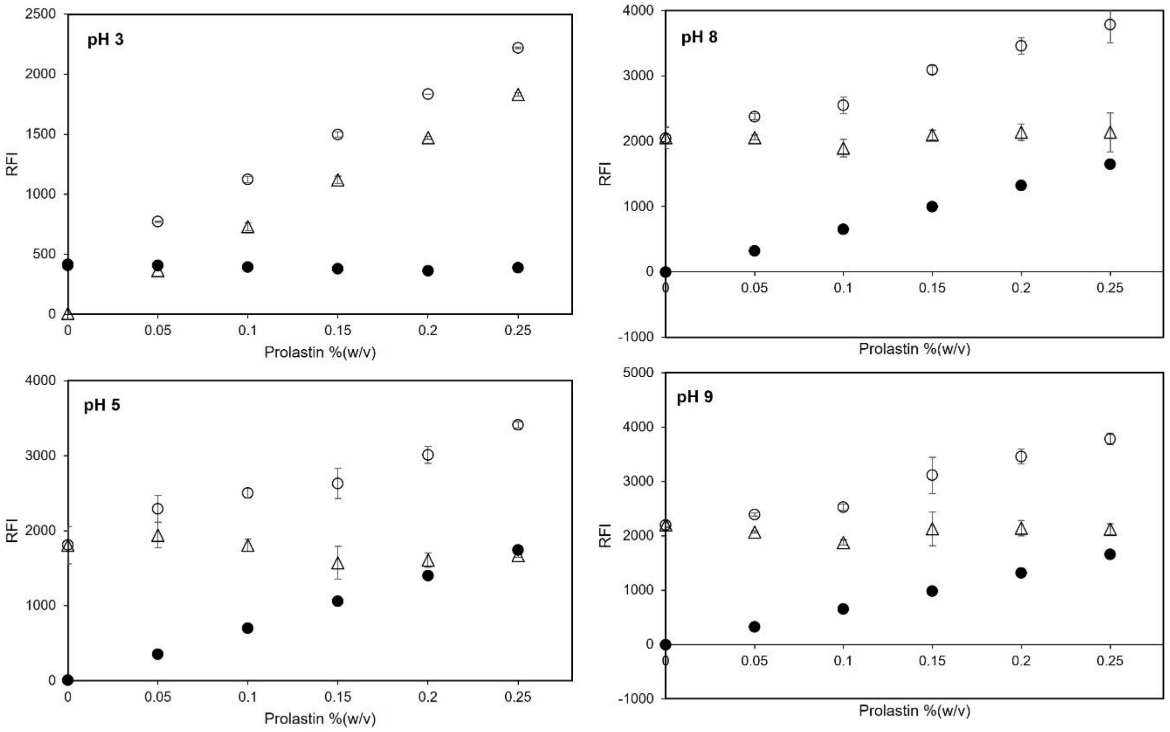

Figure S6. Relative fluorescence intensity (RFI) of Prolastin in McIlvaine buffer and 1.41 mM PRODAN in Prolastin solution versus Prolastin concentration. Open symbols (○) represent the RFI of a Prolastin solution with PRODAN. Closed symbols (●) represent the RFI of a Prolastin control solution without PRODAN. Triangles (Δ) represent the net RFI of a Prolastin solution, which is the result of subtracting the RFI of Prolastin control solution from the RFI of Prolastin solution with PRODAN. Excitation and emission wavelengths for PRODAN were 365 and 465 nm, respectively.

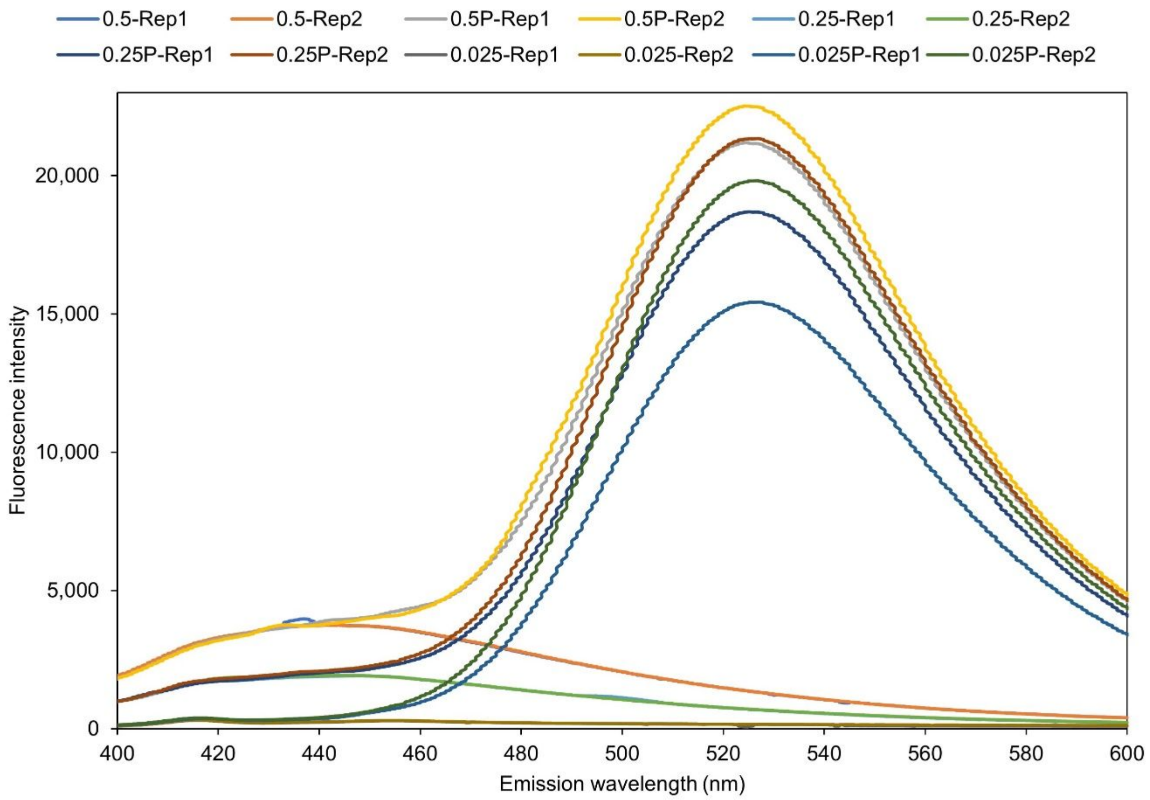

Figure S7. Fluorescence emission spectra of Prolastin control solutions at 0.025% (0.025), 0.25% (0.25) and 0.5% (0.5) (w/v) and 1.41 mM PRODAN in 0.025% (0.025P), 0.25% (0.25P) and 0.5% (0.5P) (w/v) Prolastin solutions.

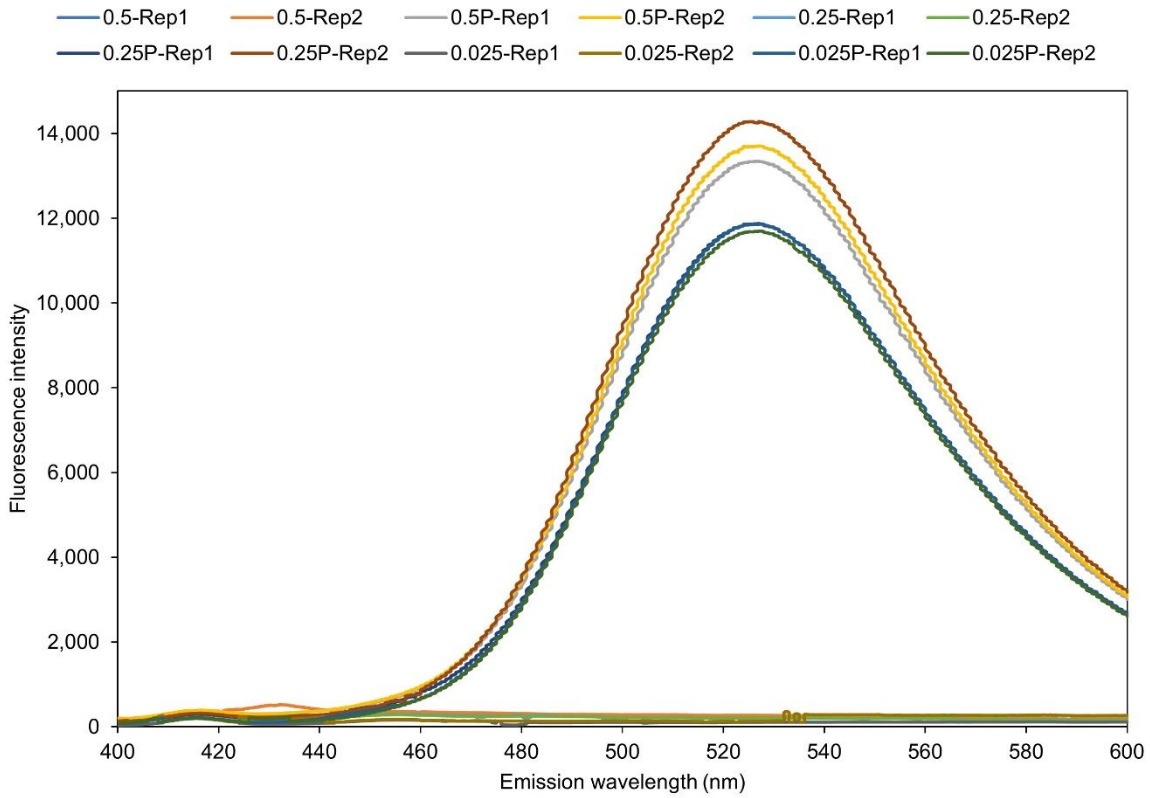

Figure S8. Fluorescence emission spectra of tryptophan control solutions at 0.025% (0.025), 0.25% (0.25) and 0.5% (0.5) (w/v) and 1.41 mM PRODAN in 0.025% (0.025P), 0.25% (0.25P) and 0.5% (0.5P) (w/v) tryptophan solutions.

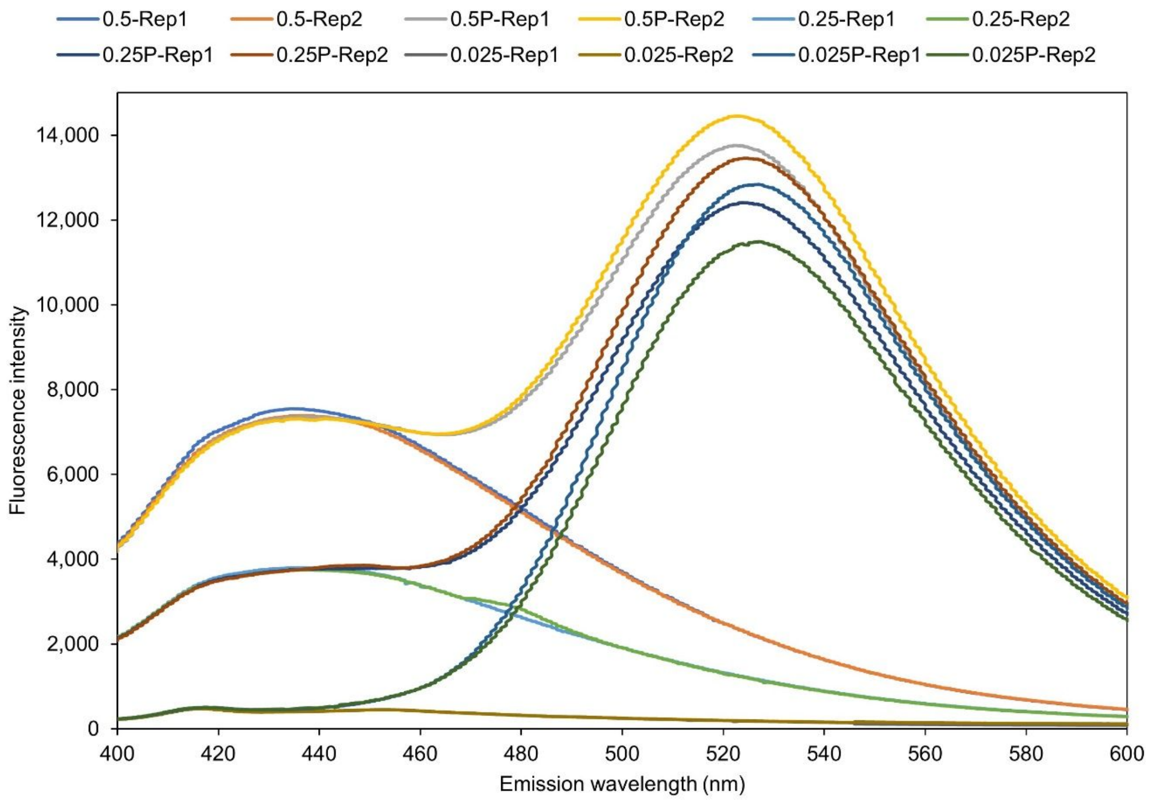

Figure S9. Fluorescence emission spectra of lysine control solutions at 0.025% (0.025), 0.25% (0.25) and 0.5% (0.5) (w/v) and 1.41 mM PRODAN in 0.025% (0.025P), 0.25% (0.25P) and 0.5% (0.5P) (w/v) lysine solutions.

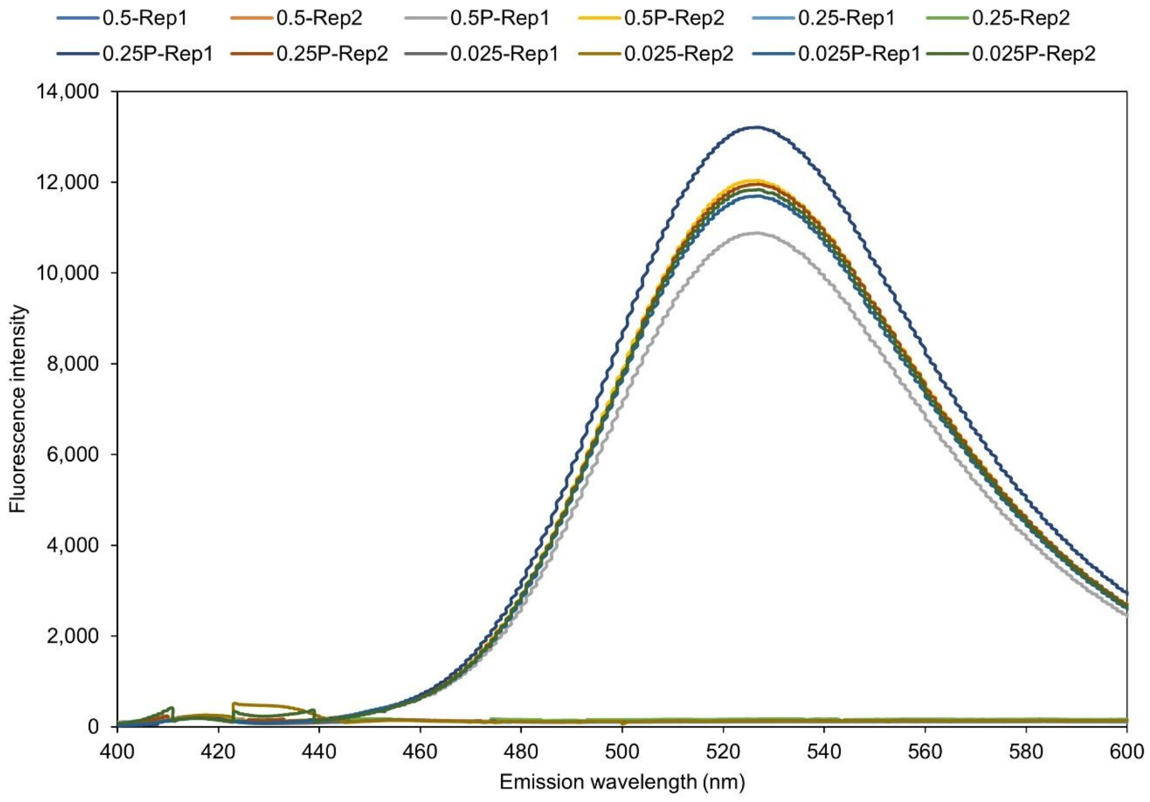

Figure S10. Fluorescence emission spectra of glutamic acid control solutions at 0.025% (0.025), 0.25% (0.25) and 0.5% (0.5) (w/v) and 1.41 mM PRODAN in 0.025% (0.025P), 0.25% (0.25P) and 0.5% (0.5P) (w/v) glutamic acid solutions.

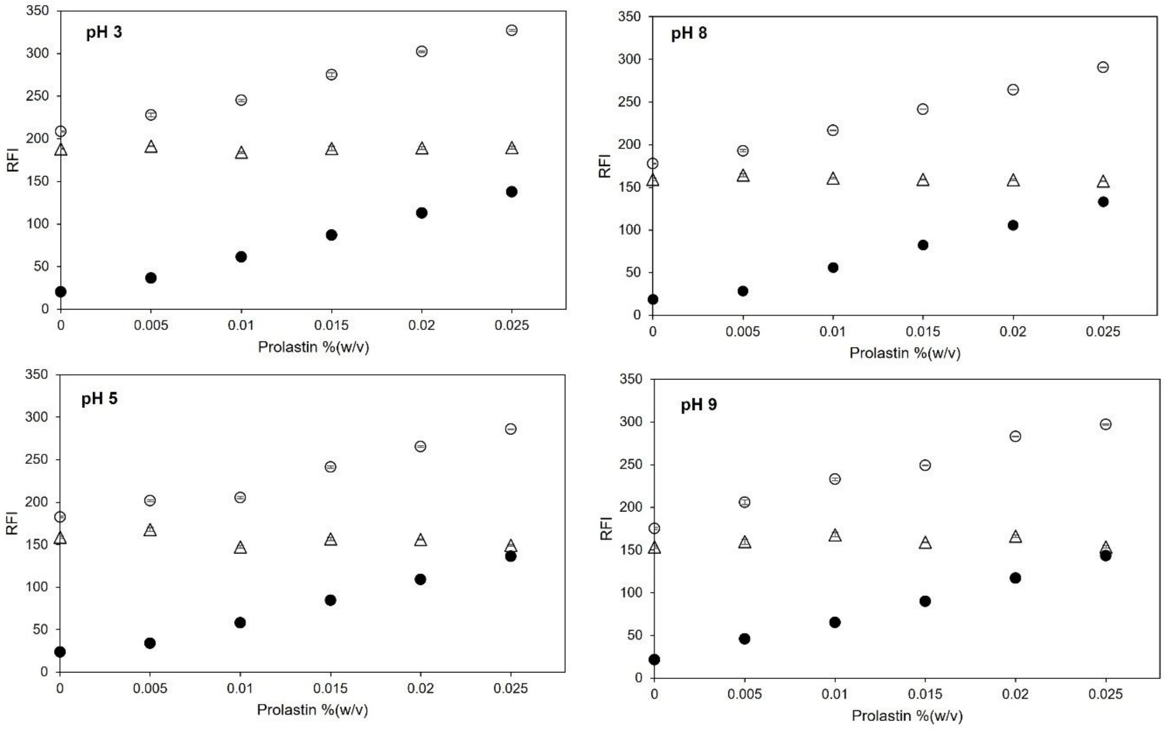

Figure S11. Relative fluorescence intensity (RFI) of Prolastin in McIlvaine buffer and 8 mM ANSA in Prolastin solution versus Prolastin concentration. Open symbols (○) represent the RFI of a Prolastin solution with ANSA. Closed symbols (●) represent the RFI of a Prolastin control solution without ANSA. Triangles (Δ) represent the net RFI of a Prolastin solution, which is the result of subtracting the RFI of Prolastin control solution from the RFI of Prolastin solution with ANSA. Excitation and emission wavelengths for ANSA were 390 and 470 nm, respectively.
